# Activation of the Endonuclease that Defines mRNA 3′ Ends Requires Incorporation into an 8-Subunit Core Cleavage and Polyadenylation Factor Complex

**DOI:** 10.1016/j.molcel.2018.12.023

**Published:** 2019-03-21

**Authors:** Chris H. Hill, Vytautė Boreikaitė, Ananthanarayanan Kumar, Ana Casañal, Peter Kubík, Gianluca Degliesposti, Sarah Maslen, Angelica Mariani, Ottilie von Loeffelholz, Mathias Girbig, Mark Skehel, Lori A. Passmore

**Affiliations:** 1 MRC Laboratory of Molecular Biology, Cambridge CB2 0QH, UK; 2 Centre for Integrative Biology, Department of Integrated Structural Biology, Institute of Genetics and of Molecular and Cellular Biology, Illkirch, Université de Strasbourg, Strasbourg, France; 3 Centre National de la Recherche Scientifique UMR 7104, Illkirch, Université de Strasbourg, Strasbourg, France; 4 INSERM U964, Illkirch, Université de Strasbourg, Strasbourg, France

**Keywords:** cleavage, polyadenylation, nuclease, mRNA, pre-mRNA, baculovirus, cryo-EM, X-ray crystallography, hydrogen-deuterium exchange, mass spectrometry

## Abstract

Cleavage and polyadenylation factor (CPF/CPSF) is a multi-protein complex essential for formation of eukaryotic mRNA 3ʹ ends. CPF cleaves pre-mRNAs at a specific site and adds a poly(A) tail. The cleavage reaction defines the 3ʹ end of the mature mRNA, and thus the activity of the endonuclease is highly regulated. Here, we show that reconstitution of specific pre-mRNA cleavage with recombinant yeast proteins requires incorporation of the Ysh1 endonuclease into an eight-subunit “CPF_core_” complex. Cleavage also requires the accessory cleavage factors IA and IB, which bind substrate pre-mRNAs and CPF, likely facilitating assembly of an active complex. Using X-ray crystallography, electron microscopy, and mass spectrometry, we determine the structure of Ysh1 bound to Mpe1 and the arrangement of subunits within CPF_core_. Together, our data suggest that the active mRNA 3ʹ end processing machinery is a dynamic assembly that is licensed to cleave only when all protein factors come together at the polyadenylation site.

## Highlights

• Crystallography and cryo-EM reveal a Ysh1-Mpe1 interface
• Specific pre-mRNA 3ʹ end cleavage is reconstituted from recombinant proteins
• Electron microscopy shows that CPF_core_ assembles around a central scaffold
• A model for activation of the 3ʹ endonuclease on substrate RNAs is proposed

## Graphical Abstract

**Figure:**
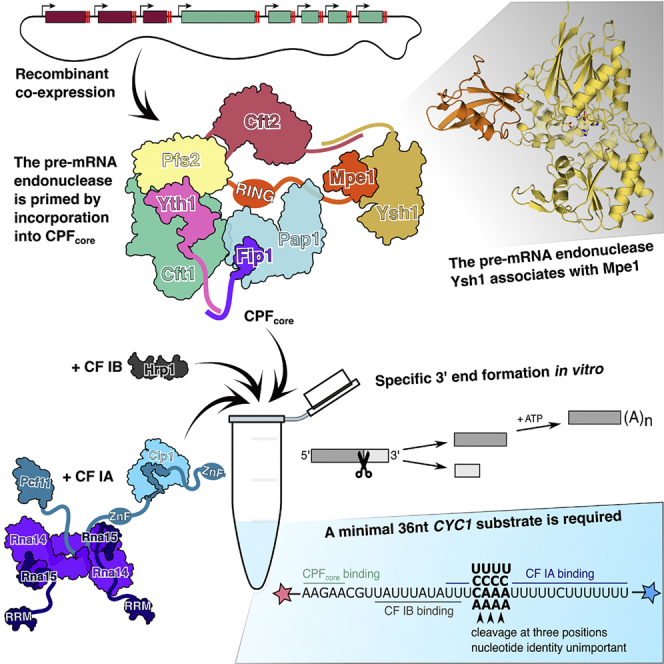

## Teaser

The 3ʹ ends of eukaryotic mRNAs are formed by endonucleolytic cleavage by Ysh1/CPSF73. Hill et al. define a minimal machinery for cleavage and polyadenylation *in vitro* using recombinant yeast proteins and short substrates. They elucidate the architecture of an ∼500 kDa eight-protein assembly, including the atomic details of a Ysh1-Mpe1 interface.

## Introduction

Eukaryotic protein-coding genes are transcribed by RNA polymerase II (Pol II) in the nucleus. The nascent pre-mRNA is capped at the 5ʹ end, spliced, and cleaved and polyadenylated at the 3ʹ end before being exported to the cytoplasm as a mature mRNA for translation. The cleavage and polyadenylation factor (CPF in yeast and CPSF in metazoans) is a large ∼1-MDa multifunctional complex with 14 different protein subunits in *Saccharomyces cerevisiae* (Casañal et al., 2017). CPF/CPSF is frequently dysregulated in viral infections and cancer (Mandel et al., 2008, Shi and Manley, 2015, Xiang et al., 2014).

To initiate pre-mRNA 3ʹ end processing and transcription termination, the nuclease enzyme Ysh1 must be correctly positioned on the pre-mRNA 3ʹ UTR and activated for cleavage. Once the cleavage reaction has occurred, the poly(A) polymerase enzyme Pap1 can access the newly generated 3ʹ-OH group to add a poly(A) tail of ∼80 nt in length (Butler and Platt, 1988). Recruitment of the Rat1 5ʹ→3ʹ exonuclease to the newly generated downstream fragment leads to Pol II termination (Kim et al., 2004). CPF also dephosphorylates serine 5 and tyrosine 1 in the C-terminal domain of Pol II to regulate transcription (Rosado-Lugo and Hampsey, 2014, Schreieck et al., 2014).

In addition to CPF, two cleavage factors (CFs) are required for efficient 3ʹ end processing: CF IA, a complex of Rna14, Rna15, Pcf11, and Clp1; and CF IB (Hrp1) (Gordon et al., 2011, Gross and Moore, 2001, Kessler et al., 1997). These essential factors bind the pre-mRNA substrate via RNA-recognition motif (RRM) domains in Rna15 and Hrp1 (Leeper et al., 2010, Pancevac et al., 2010) and zinc fingers in Pcf11 (Guéguéniat et al., 2017, Yang et al., 2017).

The RNA sequence requirements for cleavage are poorly understood. In higher eukaryotes, several *cis*-acting sequences have been identified, most notably the AAUAAA motif, located ∼10–30 nt upstream of the cleavage site (Fitzgerald and Shenk, 1981, Manley et al., 1985). The cleavage site itself is usually Y(A)_n_ (where Y is a pyrimidine) and is flanked by U-rich elements (Proudfoot, 2011). In yeast, an upstream UAUAUA “efficiency element” further enhances CPF nuclease activity (Guo and Sherman, 1996, Irniger and Braus, 1994). However, sequences directing yeast 3ʹ end formation are highly degenerate, and the above motifs are absent from many pre-mRNAs (Tian and Graber, 2012).

Mechanistic analysis of RNA recognition and nuclease activity have been historically challenging due to low purity and yield of purified CPF and the lethality of most mutants. This has been further confounded by the poor solubility of many CPF subunits in isolation and the lack of a suitable recombinant system to dissect the roles of CPF components. *In vivo* studies and reconstitution assays using extracts found that many of the CPF subunits were required for nuclease activity (Zhao et al., 1999) and that Ysh1/CPSF73 is the enzymatic component (Chanfreau et al., 1996, Dominski, 2010, Jenny et al., 1996, Ryan et al., 2004).

Ysh1 is highly conserved (53% sequence identity between the yeast and human nuclease domains). A crystal structure of the nuclease domain of human CPSF73 showed that it is comprised of a metallo-β-lactamase domain and a β-CASP domain, with the zinc-coordinated active site residing at their interface (Mandel et al., 2006). In the structure, CPSF73 is in a closed conformation with no clear path for substrate RNA to the active site.

We recently determined the overall architecture of CPF, demonstrating that the CPF subunits are organized into three functional modules based around the enzymatic activities of the complex: nuclease, polymerase, and phosphatase (Casañal et al., 2017). We used electron cryomicroscopy (cryo-EM) to study the polymerase module and found that the Cft1/CPSF160, Pfs2/WDR33, and Yth1/CPSF30 subunits are intimately associated, forming a scaffold for assembly of an active polyadenylation complex (Casañal et al., 2017). Studies of the human complex confirm that this assembly is highly conserved and recognizes the mammalian “AAUAAA” motif (Clerici et al., 2017, Clerici et al., 2018, Sun et al., 2018).

In contrast, there is little mechanistic information available on the nuclease module, which is composed of the endonuclease Ysh1/CPSF73, the pseudo-nuclease Cft2/CPSF100, and the multi-domain protein Mpe1/RBBP6 (Casañal et al., 2017). We also previously identified an alternative heterotrimeric complex of Ysh1, Mpe1, and Yjr141w/Ipa1, a protein of unknown function that is essential for yeast viability and has been implicated in polyadenylation (Casañal et al., 2017, Costanzo et al., 2016). Here, we define the interaction interfaces among Ysh1, Mpe1, and Yjr141w and show that the nuclease module alone is catalytically inactive. We demonstrate that Ysh1 is only primed for activation upon incorporation into “CPF_core_,” an eight-subunit complex. We propose a model for assembly of the CPF_core_ complex, providing insight into the mechanisms of pre-mRNA cleavage.

## Results

### The Catalytic Domain of Ysh1 Interacts Directly with the Mpe1 Ubiquitin-like Domain

**Figure 1:**
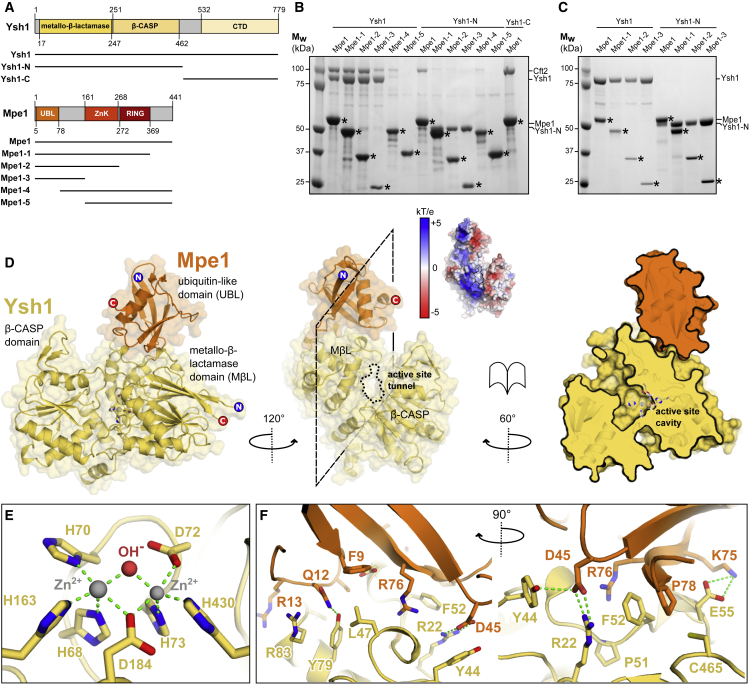
The Mpe1 UBL Domain Binds to the Ysh1 Catalytic Domain Next to the Active Site Tunnel (A) Domain diagram of Ysh1 and Mpe1 proteins, with truncations indicated by black lines. ZnK, zinc knuckle. (B) SDS-PAGE analysis of pull-down experiments following baculovirus-driven co-expression of pairs of Ysh1 and Mpe1 constructs shown in (A), with full-length Cft2. The tagged Mpe1 constructs (asterisks) were captured by Strep-Tactin resin, and co-purification of Ysh1 and Cft2 was analyzed. (C) SDS-PAGE analysis of complexes identified in (B) after anion exchange chromatography. The Ysh1-Mpe1 proteins remain associated, but Cft2 dissociates. (D) X-ray crystal structure of the Ysh1 N-terminal catalytic domain (yellow) bound to the Mpe1 N-terminal UBL domain (orange). N and C termini of both models are indicated, zinc-coordinating residues are shown in sticks, and zinc ions are spheres. A slice through the complex (right) reveals a narrow tunnel leading to a large solvent-filled cavity adjacent to the active site. Inset: electrostatic surface potential at pH 7.4. A large basic patch comprising residues from both proteins lies adjacent to the active site tunnel. (E) Details of metal ion coordination in the Ysh1 active site. (F) Details of the interface between Ysh1 and Mpe1. Hydrogen bonds and electrostatic interactions are indicated by green dashed lines. Two orthogonal views are shown. See also Figure S1.

To understand the assembly and structure of the CPF nuclease module, we attempted to express and purify a Ysh1-Mpe1-Cft2 complex. Although these subunits have direct contacts within native CPF (Casañal et al., 2017), the recombinant nuclease module was not stable in solution; Cft2 dissociated during anion exchange or size exclusion chromatography, leaving a dimeric Ysh1-Mpe1 complex. To characterize the Ysh1-Mpe1 interaction, we made a series of Ysh1 and Mpe1 domain truncations (Figure 1A) and co-expressed these in insect cells, along with Cft2. A StrepII-tag on Mpe1 was used to pull down interacting components from cell lysates. Full-length Mpe1 co-purified with Ysh1, but after removal of residues 1–78 (Mpe1-4) or 1–161 (Mpe1-5), this interaction could no longer be detected (Figure 1B). This N-terminal region of Mpe1 that is required for Ysh1 interaction contains a ubiquitin-like (UBL) domain.

In the pull-down assays, Mpe1 interacted with full-length Ysh1 and the catalytic N-terminal domain (Ysh1-N), but not with the C-terminal domain (Ysh1-C) (Figure 1B). Removal of the Ysh1 C-terminal domain and parts of Mpe1 both reduced the interaction with Cft2. Together, these data suggest that the N-terminal catalytic domain of Ysh1 interacts with the Mpe1 UBL domain. To further assess the stability of these interactions, complexes identified by pull-down were subjected to anion exchange chromatography. The interactions between Ysh1 and Mpe1 constructs were stable, whereas Cft2 dissociated during purification (Figure 1C), consistent with Cft2 dissociation in earlier attempts to purify the Ysh1-Mpe1-Cft2 complex.

### Structure of Ysh1 Catalytic Domain Bound to Mpe1 UBL

To investigate the molecular details of the Ysh1-Mpe1 interaction, we determined the X-ray crystal structure of a complex between the catalytic domain of Ysh1 (residues 1–473) and the UBL domain of Mpe1 (residues 1–120). The structure was refined to 2.3 Å resolution (Table 1). Ysh1 adopts a globular fold comprised of a metallo-β-lactamase and β-CASP domain (Figure 1D), similar to the human 3ʹ endonuclease CPSF73 and yeast Cft2 (Figure S1A) (Mandel et al., 2006). Density was visible for a β-CASP α helix (residues 290–310) that was disordered in the CPSF-73 structure (Figures S1A and S1B). A loop between residues 114 and 126 was disordered in both structures and could not be modeled.

**Table 1:** Crystallographic Data Collection, Processing, and Refinement

**Data collection**	

Space group	P2_1_
a, b, c (Å)	43.38, 124.27, 63.45
α, β, γ (°)	90.0, 103.21, 90.0
Number of reflections	99,955 (4,970)
Resolution range (Å)	62.13–2.28 (2.32–2.28)
Completeness (%)	99.08 (99.38)
Redundancy	3.39 (3.45)
〈I/σ(I)〉^a^	12.3 (1.3)
CC_1/2_	0.999 (0.578)
R_merge_	0.065 (1.13)

**Refinement**	

Resolution range (Å)	62.13–2.28
Number of reflections in working set	27,979 (2510)
Number of reflections in free set	1,487 (144)
R_work_/R_free_	0.1726/0.2219
Number of atoms	4,565
Average B-factors (Å^2^)	72.2
Ramachandran	
Favored (%)	95.91
Outliers (%)	0.37
RMSDs	
Bonds (Å)	0.003
Angles (**°**)	0.56

Values for the outer shell are given in parentheses.

a Mean I/σ(I) is >2.0 at resolutions >2.5 Å. The CC_1/2_ values (above) were used to decide resolution cutoff (Karplus and Diederichs, 2012).

Residues H68, H70, D72, H73, H163, D184, and H430 comprise the active site, coordinating two Zn^2+^ ions with octahedral geometry (Figure 1E). A water molecule occupies the position for the activated hydroxyl nucleophile between the two metal ions (Mandel et al., 2006). The catalytic core is located in a large internal solvent-filled cavity at the boundary between the metallo-β-lactamase and β-CASP domains, with a narrow tunnel leading to the surface of the enzyme (Figure 1D).

The UBL domain of Mpe1 consists of a central α helix flanked by a curved 4-stranded β sheet and capped by an additional pair of short anti-parallel β strands, in the same configuration as the UBL domain of the human ortholog RBBP6 (root mean square deviation [RMSD] = 1.36 Å over 452 atoms; (Pugh et al., 2006)). Beyond that, residues 81–98 and 108–120 are disordered, and C-terminal residues 99–107 form a short helical turn that packs against the central α helix.

The interface between Mpe1 and Ysh1 buries an area of ∼900 Å^2^ and involves hydrophobic, polar, and electrostatic contacts between Mpe1 residues in loops and β strands and Ysh1 residues on the top surface of the metallo-β-lactamase domain (Figures 1F and S1C). Docking experiments suggest that this interface may be conserved in human CPSF73 and RBBP6 (Figures S1D and S1E).

Mpe1 binds to the Ysh1 metallo-β-lactamase domain next to the active site tunnel opening. A large basic patch is formed by contiguous surfaces of both Ysh1 and Mpe1, suggesting a possible role in RNA binding (Figure 1D). However, in the structure, Ysh1 remains in a “closed” conformation that is unlikely to be catalytically active because the active site tunnel is too narrow to accommodate the entire RNA substrate. Atomic B factors indicate that the metallo-β-lactamase domain is more ordered than the β-CASP domain, implying that movement within the latter may activate the enzyme by further opening the tunnel to the active site (Figure S1B). Compared to the CPSF73 structure, two helices in the metallo-β-lactamase domain are shifted toward the Mpe1 binding site (Figure S1A), consequently widening the cleft between the two Ysh1 domains. Such a movement could be a precursor to full Ysh1 activation.

### Mpe1 and Yjr141w Bind Independently to Ysh1 at Distinct Sites

**Figure 2:**
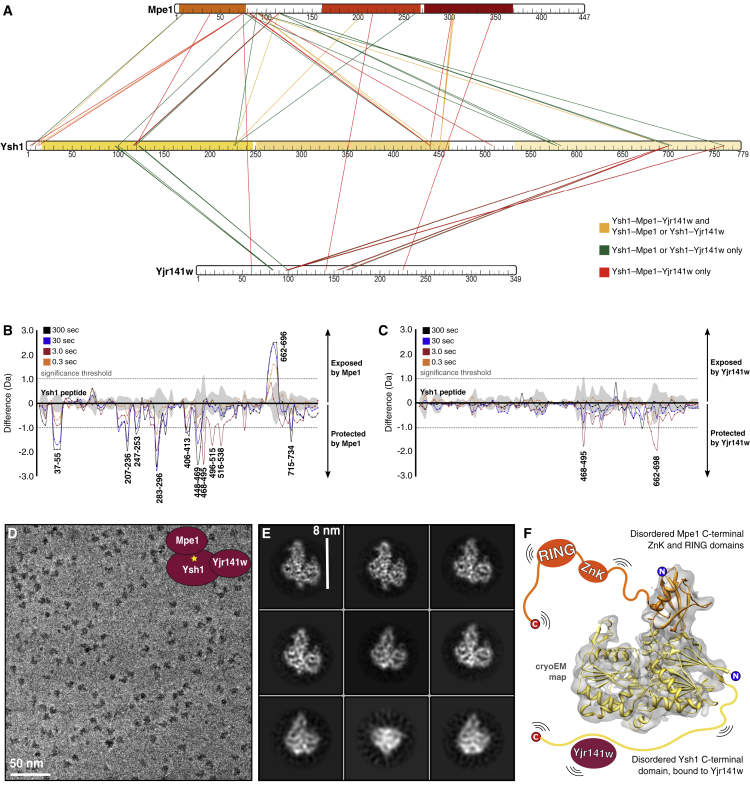
Mass Spectrometry and Cryo-EM Define the Interactions among Full-Length Ysh1, Mpe1, and Yjr141w (A) Interactions among Ysh1, Mpe1, and Yjr141w mapped by crosslinking mass spectrometry of the Ysh1-Mpe1-Yjr141w trimer and Ysh1-Mpe1 and Ysh1-Yjr141w heterodimers. Lines are color-coded as indicated. (B) Hydrogen-deuterium exchange mass spectrometry difference plot (Ysh1-Mpe1-Yjr141w versus Ysh1-Yjr141w) showing peptides of Ysh1 that are protected (negative) and exposed (positive) by Mpe1. (C) Hydrogen-deuterium exchange mass-spectrometry difference plot (Ysh1-Mpe1-Yjr141w versus Ysh1-Mpe1) showing peptides of Ysh1 that are protected (negative) and exposed (positive) by Yjr141w. In (B) and (C), triplicate data from four independent color-coded time-points are shown. The significance threshold is indicated by a dotted line. Gray shading indicates the SD of all charge states and replicates per peptide. (D) Cryo-EM analysis of the Ysh1-Mpe1-Yjr141w heterotrimer. A representative micrograph at original magnification × 105,000 and −0.5 μm defocus. (E) Selected 2D class averages of aligned particles. (F) The crystal structure from Figure 1D was docked into the EM map filtered to 6 Å resolution. No density was observed for the Ysh1 CTD or the Yjr141w or Mpe1 CTDs. See also Figure S2 and Table S1.

In addition to the Ysh1-Mpe1 complex, both the Ysh1-Yjr141w dimer and the trimeric Ysh1-Mpe1-Yjr141w complex could be purified and were stable in solution. To map the Mpe1 and Yjr141w binding sites on Ysh1 in more detail, these complexes were chemically crosslinked then analyzed by mass spectrometry (XL-MS). Many of the observed crosslinks were between Mpe1 N-terminal residues 1–120 and the N-terminal catalytic domain of Ysh1 (1–462), consistent with the crystal structure, but crosslinks were present throughout the Ysh1 sequence (Table S1; Figure 2A). In contrast, Yjr141w was predominantly crosslinked to Ysh1 residues 680–779 in the C-terminal domain (Table S1; Figure 2A). Very few crosslinks between Yjr141w and Mpe1 were observed, suggesting that these proteins do not directly interact.

We also analyzed these complexes by hydrogen-deuterium exchange mass spectrometry (HDX-MS; Figure S2A). By comparing the rate of deuterium incorporation into Ysh1 peptides in the presence and absence of binding partners, it is possible to identify Ysh1 regions that become protected or exposed upon Mpe1 or Yjr141w binding (Figures S2B and S2C). These analyses indicate that Mpe1 interacts with the N-terminal catalytic domain of Ysh1 at several sites, primarily at residues 37–55 and 207–469 (Figure 2B), while Yjr141w interacts with the Ysh1 C-terminal domain at residues 468–495 and 662–698 (Figure 2C). These observations are consistent with the crosslinking experiments (Figure 2A).

The relative fractional uptake of deuterium also provides an indication of the disorder of any given peptide. This showed that in the absence of other CPF subunits, the only well-ordered regions of the three proteins analyzed were the N-terminal catalytic domain of Ysh1 and the N-terminal UBL and C-terminal RING domains of Mpe1 (Figures S2B–S2E). Together, HDX and XL-MS data validated the interactions that we observed in the crystal structure and also highlighted regions of Ysh1 that may bind to other parts of Mpe1 (Figures S2F and S2G).

### Cryo-EM of a Ysh1-Mpe1-Yjr141w Complex Reveals Extensive Flexibility

To further investigate the Ysh1-Mpe1 interaction and to determine how Yjr141w associates with Ysh1, we studied the 177-kDa Ysh1-Mpe1-Yjr141w complex by cryo-EM (Table 2; Figure 2D). The 2D class averages resembled the crystal structure (Figure 2E) but a strongly preferred orientation limited the overall resolution of our 3D reconstruction (Figures 2F and S2H–S2J). Still, when filtered to 6.0 Å, alpha helices were clearly identified allowing us to reliably place our crystal structure into the cryo-EM map. Interestingly, the only component of the trimer that aligned well and contributed to the 3D structure was the 57-kDa complex between the Ysh1 catalytic domain and the Mpe1 UBL domain that we had crystallized. In our cryo-EM maps, none of the additional Ysh1 regions identified by HDX as potential Mpe1 binding surfaces were observed to make stable structural contacts with Mpe1, and Yjr141w was not visible.

**Table 2:** EM Data Collection and Processing

	Ysh1-Mpe1-Yjr141w	CPF_core_		CPF_pol_ + Cft2
	Cryo-EM	Cryo-EM	Negative-Stain EM	Negative-Stain EM
Data collection				

Microscope	Titan Krios	FEI Tecnai Polara	FEI Tecnai Spirit	FEI Tecnai Spirit
Detector	K2	Falcon III	Ultrascan 1000	Ultrascan 1000
Magnification	105,000 ×	59,000 ×	26,000 ×	26,000 ×
Pixel size (Å)	1.09	1.78	3.98	3.98
Voltage (keV)	300	300	120	120
Electron dose (e-/Å^2^)	∼45	∼60	∼40–60	∼40–60
Defocus range (μm)	−0.5 to −0.7	−2.5 to −4.5	−0.6	−0.6
Phase shift range (°)^a^	20–140	N/A	N/A	N/A
Number of particles	43,308	120,773	23,969	38,142

Processing				

Resolution	4.8	N/A^b^	20	N/A^b^
Efficiency (E_od_)^c^	0.29	N/A^b^	0.79	N/A^b^

N/A, not available.

a Volta phase plate used during data collection.

b 3D reconstruction not performed.

c Naydenova and Russo (2017).

Taken together, our crystallography, cryo-EM, and mass spectrometry data show that the UBL domain of Mpe1 binds to the N-terminal catalytic domain of Ysh1. The remainder of Mpe1 appears to be flexible in the absence of other binding partners. The C-terminal domain of Ysh1 interacts with Yjr141w and Cft2 but is also flexible or disordered in the Ysh1-Mpe1-Yjr141w trimeric complex (Figure 2F).

### Ysh1 Is Primed for Activation by Assembly into an Eight-Subunit CPF_core_ Complex

**Figure 3:**
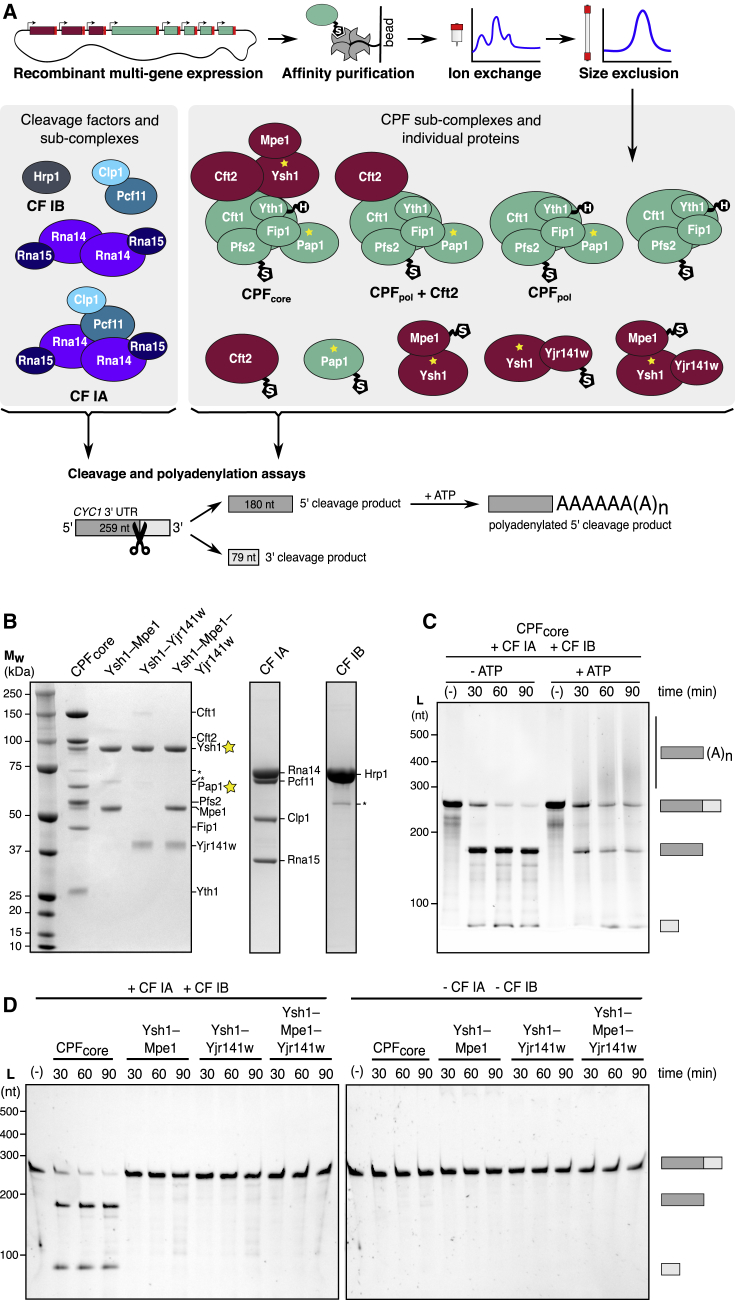
Ysh1 Is Primed for Activation by Assembly into an Eight-Subunit CPF_core_ Complex (A) Schematic diagrams showing the expression and purification workflow, composition of recombinant complexes, and details of the *in vitro* activity assay. Proteins are represented by circles, with a yellow star to highlight an enzymatic subunit. S, StrepII tag; H, His_6_ tag; CPF_pol_, polymerase module. (B) SDS-PAGE analysis of recombinant protein complexes after affinity, anion exchange, and size exclusion chromatography. Asterisks indicate contaminant proteins. (C) The *CYC1* model pre-mRNA is specifically cleaved by CPF_core_ with CF IA and CF IB, and the 5ʹ-cleavage product is polyadenylated in the presence of ATP, as shown by denaturing gel electrophoresis of RNA. The negative control reaction (−) contained CF IA and CF IB, but not CPF_core_. (D) Denaturing RNA gel electrophoresis of cleavage assay time courses performed using the protein complexes shown in (B). The negative control lanes (−) show no RNA cleavage when incubated with CF IA and CF IB (left) or buffer (right) for 90 min. See also Figure S3.

When we tested the activity of the dimeric and trimeric Ysh1-containing complexes, we found that they were not active in cleavage assays (see below), consistent with the closed conformation observed in the Ysh1-Mpe1 crystal structure (Figure 1D). Other CPF subunits may be required for Ysh1 activity and its stable incorporation into larger complexes. Thus, to determine the requirements for Ysh1 activation, we created a series of baculovirus constructs to produce different subcomplexes of CPF (Figures 3A and 3B). We were able to purify a stable, Ysh1-containing complex comprising all subunits from the nuclease (Ysh1, Cft2, and Mpe1) and polymerase (Cft1, Pfs2, Yth1, Fip1, and Pap1) modules; we refer to this eight-protein assembly as CPF_core_. We also purified cleavage factors CF IA and CF IB and their subcomplexes (Figures 3A and 3B).

First, we tested the ability of CPF_core_ to perform coupled cleavage and polyadenylation. We used the 259-nt 3ʹ UTR of the *CYC1* transcript as a model pre-mRNA substrate (Butler and Platt, 1988). In the presence of CF IA and CF IB, CPF_core_ specifically cleaved *CYC1* RNA into two products of the expected size and added a poly(A) tail to the upstream fragment (Figure 3C). To determine which subunits were required for cleavage activity, we tested each of the smaller Ysh1-containing complexes, but none of them were active (Figure 3D). The requirement for Ysh1 assembly in an ∼0.5-MDa complex explains why specific CPF endonuclease activity had not previously been demonstrated with recombinant components.

CF IA and CF IB were required for efficient RNA cleavage by CPF_core_ (Figure 3D), consistent with their essential roles in 3′ end formation; CPF_core_ had very weak but specific nuclease activity without CF IA and CF IB (Figure S3A). CF IA alone (but not CF IA subcomplexes) activated CPF_core_ to cleave at the correct site, but it also promoted cleavage at a secondary site within the upstream 5′ cleavage product (Figure S3B). In contrast, CF IB alone activated cleavage weakly but at the correct site. Thus, CF IA activates cleavage while CF IB enforces specificity and prevents secondary cleavage events.

CPF_core_ is produced by co-expression of its constituent subunits from one multi-gene baculovirus construct. We also attempted to reconstitute an active nuclease complex by mixing together equimolar amounts of CPF_core_ subunits or subcomplexes that had been expressed and purified separately. We incubated these with the *CYC1* substrate and found that none of these *in vitro* reconstituted complexes were active (Figure S3C). Addition of purified Yjr141w to CPF_core_ also had no substantial effect on pre-mRNA cleavage *in vitro* (Figure S3D). This suggests that an *in vivo* assembly pathway for CPF_core_ is critical for nuclease activation.

CPF_core_ contains 8 of the 14 CPF subunits. To determine whether the missing phosphatase module subunits (Pta1, Glc7, Ref2, Swd2, Pti1, and Ssu72) contribute to cleavage, we compared the activity of recombinant CPF_core_ to endogenous CPF purified from yeast. The nuclease activity and specificity of endogenous CPF were very similar to that of CPF_core_ (Figure S3E), suggesting that the phosphatase module does not substantially contribute to RNA recognition or nuclease activation *in vitro*.

### Cleavage by CPF_core_ Requires a 36-nt Sequence within the *CYC1* 3ʹ UTR

**Figure 4:**
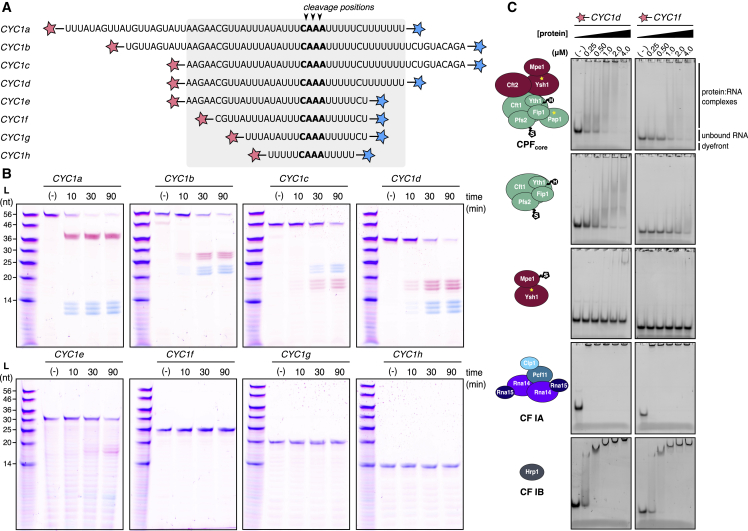
CPF_core_ Binds and Cleaves a 36-nt Minimal RNA Substrate (A) Sequences of RNA substrates derived from the *CYC1* 3ʹ UTR. Each substrate carries both 5ʹ-FAM and 3ʹ-A647 labels (red and blue stars, respectively). The canonical cleavage site is highlighted in bold, and the minimal sequence required for efficient cleavage is represented by the gray box. (B) Denaturing gel electrophoresis of the short RNA substrates after incubation with CPF_core_, CF IA, and CF IB. The negative control reaction (−) contained CF IA and CF IB, but not CPF_core_. (C) Electrophoretic mobility shift assays (EMSAs) performed with *CYC1d* (cleaved by CPF_core_) and *CYC1f* (not cleaved by CPF_core_) RNAs. See also Figures S4–S6 and Table S2.

To determine which regions of the 259-nt *CYC1* 3ʹ UTR are necessary for endonucleolytic cleavage by CPF_core_, we designed a series of 5ʹ and 3ʹ truncations around the known cleavage site (Figure 4A). These short RNA substrates were synthesized with different fluorescent labels on each end, allowing visualization of both 5ʹ and 3ʹ cleavage products with single-nucleotide resolution following denaturing gel electrophoresis (Figure S4A).

Substrates *CYC1a*, *CYC1b*, *CYC1c*, and *CYC1d* were cleaved efficiently by CPF_core_ (Figure 4B). Further removal of UUUUU from the 3ʹ end or AAGAA from the 5ʹ end reduced (*CYC1e*) or abolished (*CYC1f*, *CYC1g*, and *CYC1h*) RNA cleavage by CPF_core_.

Despite the different lengths, every RNA substrate was cut at three positions within the same CAAA motif (Figure S4A), and there was no evidence of exonuclease activity. Interestingly, the cleavage event is specific but equally likely to occur at any of the positions within this motif. This 3-nt window was extended to 5 nt if cleavage was slowed by introducing C-A-A-A- phosphothioate bonds (Figures S4B and S4C). Mutation of the CAAA to GAAA, UAAA, or AAAA within the 36-nt *CYC1d* RNA did not abolish cleavage (Figure S4C). Thus, we made even more drastic mutations of the CAAA cleavage site to CCCC, GGGG, or UUUU. CPF_core_ cleaved the *CYC1d*-CCCC substrate but had very weak activity on *CYC1d*-GGGG (Figure S4D). *CYC1d*-UUUU RNA was cleaved with reduced activity, and the cleavage window was expanded even further to 6 nt, possibly because the resultant U_12_ is a slippery sequence. Together, this suggests that endonucleolytic cleavage is not limited to a specific nucleotide identity, and once Ysh1 is activated, it cleaves the bound RNA within a positional window.

### RNA Recognition Requires Complementary Binding Properties of CPF_core_ and Cleavage Factors

To determine which subunits of CPF and cleavage factor are involved in recognition of the minimal pre-mRNA substrate that is efficiently processed *in vitro*, we conducted a series of electrophoretic mobility shift assays (EMSAs). Each of the proteins and subcomplexes that could be stably purified (Figure 3A) were tested for binding to A_15_, U_15_, C_15_, G_15_, *CYC1d* (cleaved by CPF_core_), and *CYC1f* (not cleaved by CPF_core_) RNAs (Figures 4C and S5; Table 3). CF IA bound to RNA with the highest affinity, showing preference for the short *CYC1* substrates as well as U_15_ and G_15_ sequences. CF IB bound every sequence with moderate affinity except C_15_.

**Table 3:** Summary of EMSA Experiments (Figure S5) Testing All Stable Components and Subcomplexes for RNA Binding Activity

Protein or complex	RNA					
	A_15_	U_15_	C_15_	G_15_	*CYC1d*	*CYC1f*
CF IB	+	++	−	+++	++++	++++
CF IA	−	+++++	−	+++++	+++++	+++++
Pcf11-Clp1	−	−	−	−	+++	++
Rna14-Rna15	−	+++	−	+++	+++	++
Cft2	−	−	−	+++	+++	++
Pap1	−	−	−	−	−	−
Ysh1-Mpe1-Yjr141w	−	−	−	+++	+	+
Ysh1-Mpe1	−	−	−	+++	+	+
Ysh1-Yjr141w	−	−	−	−	−	−
CPF_pol_ (no Pap1)	++	−	−	++++	+++	+
CPF_pol_	++	−	−	++++	+++	+
CPF_pol_ + Cft2	++	−	−	+++++	++++	++
CPF_core_	++	−	−	+++++	++++	++

*CYC1d*, *CYC1f*, and 15-mers of A, U, C, and G were used.

CPF_core_ bound to *CYC1d* with slightly higher affinity than to *CYC1f* (Figure 4C). Interestingly, the polymerase module exhibited a similar binding pattern. Both CPF_core_ and the polymerase module also bound to A_15_ and G_15_. Cft2 bound to both *CYC1* RNAs and G_15_. In contrast, RNA binding by Pap1, Ysh1, and Yjr141w was not detectable, while Mpe1 bound to only G_15_ (Figure S5; Table 3).

Assembly of the active 3′ end processing machinery likely involves formation of multiple protein-RNA and protein-protein interactions. Pull-down experiments confirmed that CF IA and Rna14–15 bound tightly to complexes containing the polymerase module (with and without Pap1) (Casañal et al., 2017). However, these did not reveal any additional interactions between CPF_core_ and the cleavage factors (Figure S6).

No components of the CPF_core_ bound to U_15_, and none of the complexes or proteins tested bound strongly to C_15_. This allowed us to exclude the machinery from binding to specific regions of our minimal *CYC1d* substrate by replacing the sequence of interest with poly(C). Mutating the 5ʹ AAGAA to CCCCC completely blocked cleavage by CPF_core_, similar to the effect of truncating it in *CYC1f* (Figures 4B and S4D). Changing the 3ʹ UUUUUU to CCCCCC reduced cleavage activity but had a somewhat milder effect than truncating it in *CYC1e.* However, nuclease activity was inhibited by replacing the entire sequence downstream of the canonical cleavage site with a C_16_ stretch (Figure S4E). Taken together, these data suggest a model in which the 5ʹ AAGAA of the *CYC1* model RNA is bound by the polymerase module within CPF_core_, while CF IA and CF IB bind to U-rich sequences.

### Ysh1, Cft2, and Pap1 Are Peripheral to the Scaffold of the Polymerase Module

**Figure 5:**
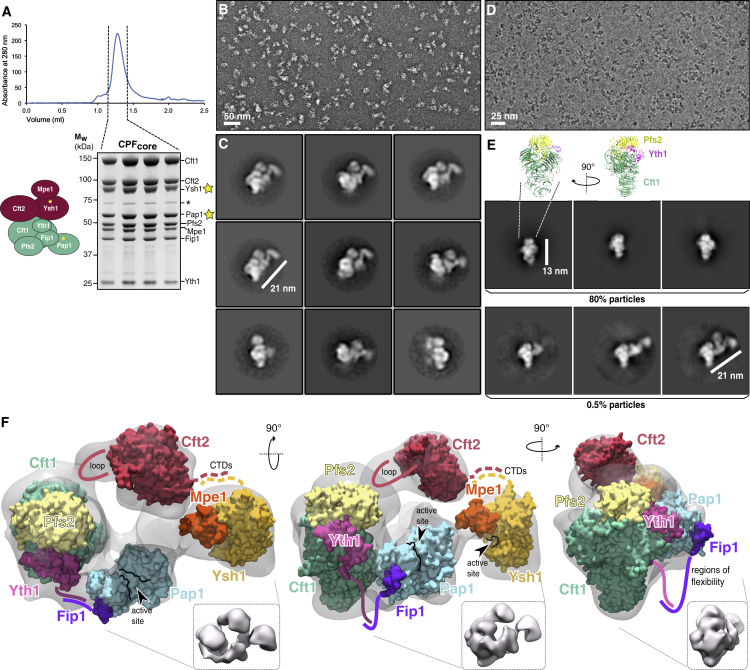
The Enzymatic Subunits of CPF_core_ Assemble around a Central Scaffold (A) Size-exclusion chromatography of CPF_core_ and SDS-PAGE analysis of fractions across the peak. Asterisks indicate contaminant proteins. (B) Representative negative-stain micrograph of CPF_core_. (C) Negative-stain 2D class averages show a distinctive 21-nm particle with the polymerase module at one end. (D) Representative cryo-EM micrograph of CPF_core_. (E) Selected 2D class averages from cryo-EM analysis of CPF_core_. Approximately 80% of the particles are present in classes that comprise the 13-nm scaffold of the polymerase module only. Up to three additional subunits are visible in ∼0.5% of the particles. (F) A model for the structure of CPF_core_ obtained from a 3D reconstruction of the negative-stain data. Three orthogonal views filtered to 25 Å are shown as insets. The cryo-EM structure of Cft1-Pfs2-Yth1 (Casañal et al., 2017) and X-ray crystal structures of Cft2 (Mandel et al., 2006), Pap1-Fip1 (Meinke et al., 2008), and Ysh1-Mpe1 (this work; Figure 1) are docked into the negative-stain map. Known disordered or flexible regions are indicated with colored lines. The weak interaction between Ysh1 and Cft2 CTDs is indicated with dashed lines. Also see Figure S7.

To gain insight into how Ysh1 is activated on incorporation into the eight-subunit assembly, we used EM to study the structure of CPF_core_. This complex was stable and mono-disperse after size-exclusion chromatography (Figure 5A). Analysis of a chemically cross-linked complex by negative-stain EM showed well-separated particles (Figure 5B). 2D class averages revealed a distinctive ∼21-nm particle with the Cft1-Pfs2-Yth1 scaffold of the polymerase module at one end and three globular densities extending from it (Figure 5C).

2D class averages from cryo-EM micrographs revealed similar structures with one, two, or three globular densities next to the scaffold, with the same dimensions and spatial arrangement as the negative-stain particles (Figures 5D and 5E, Table 2); however, these classes originated from only ∼0.5% of particles. Instead, the cryo-EM dataset was dominated by smaller particles representing the Cft1-Pfs2-Yth1 scaffold. This suggests that the other globular densities corresponding to Cft2 and Pap1 are either highly flexible or dissociate during specimen preparation, despite chemical crosslinking.

Using the negative-stain dataset, we obtained a 3D reconstruction of CPF_core_ at ∼20 Å resolution. We built a model for CPF_core_ by docking known X-ray crystal structures and cryo-EM structures into the map (Figure 5F). In our model, the Cft1-Pfs2-Yth1 scaffold with its four characteristic β propellers is located at one end of the complex with Pap1 in close proximity. Pap1 is known to be tethered to the complex through Fip1, which in turn binds zinc fingers 4 and 5 of Yth1 (disordered in the docked cryo-EM structure) (Barabino et al., 2000, Helmling et al., 2001, Meinke et al., 2008, Tacahashi et al., 2003).

We modeled the largest globular density proximal to the scaffold as the metallo-β-lactamase/β-CASP domain of the pseudo-nuclease Cft2. A disordered loop of 204 amino acids, absent from the crystal structure of Cft2, could act as a flexible tether. Ysh1 is distal to the scaffold of the polymerase module in our model and is oriented by both Cft2 and Mpe1, as validated by analysis of CPF_core_ lacking Ysh1 and Mpe1 (Figure S7A). We juxtaposed the disordered C termini of Cft2 and Ysh1 in our model based on our pull-down data and evidence that the C-terminal domains of human orthologs (CPSF100 and CPSF73) interact (Dominski et al., 2005). These domains may be located within the density extending between Mpe1 and Pap1. Alternatively, this may be the Mpe1 zinc knuckle or RING domains.

## Discussion

Pre-mRNA cleavage is the decisive event in mRNA 3ʹ end formation and transcription termination; a poly(A) tail cannot be added until the 3ʹ-OH of the upstream product is released, and the downstream cleavage product is required for the Rat1 5ʹ→3ʹ exonuclease to trigger Pol II termination (Kim et al., 2004). Here, using a fully recombinant approach, we show that an eight-subunit CPF_core_ complex, CF IA, and CF IB represent the minimal machinery for 3ʹ end formation *in vitro*. Strikingly, it appears that the phosphatase module of CPF (Casañal et al., 2017, Nedea et al., 2003) is dispensable in this fully reconstituted *in vitro* system with purified proteins. This suggests that within CPF, Pol II regulatory functions that are essential *in vivo* (or in cell-extract systems) are separable from pre-mRNA substrate processing, reinforcing the functional distinction between CPF enzymatic modules (Casañal et al., 2017).

### Priming of the Ysh1 Endonuclease

For the cleavage event to occur, a mechanism must exist to open the Ysh1 active site channel, widening the cleft between the metallo-β-lactamase and β-CASP domains to allow substrate RNA to access the catalytic center. This is likely highly regulated to prevent spurious, nonspecific cleavage of cellular RNAs before Ysh1 is incorporated into CPF. The need for such regulation is emphasized by our observation that once activated, Ysh1 itself displays little sequence specificity. Off-target nuclease activity is minimized, because the Ysh1-Mpe1 complex exists in an inactive, autoinhibited state. In addition, Ysh1 has a relatively low binding affinity for RNA (Table 3), and the Ysh1-Mpe1 complex does not strongly interact with cleavage factors (Figure S6). We propose that the correct assembly of Ysh1 into CPFcore is essential to “prime” the nuclease for activation. In this primed state, Ysh1 within CPF_core_ displays specific activity, but at very low levels (Figure S3A).

Since mixing separately purified subunits together does not result in an active complex, it is likely that the assembly of CPF_core_ is also a regulated process. For example, activation may require a co-translational assembly mechanism in which unstructured regions fold together. Alternatively, post-translational modification, chaperone activity, or cofactor binding may be required. Yjr141w is a candidate assembly factor, as it binds to the Ysh1 C-terminal domain and prevents aggregation, but it is not a component of CPF_core_ and does not appear to directly affect nuclease activity. Yjr141w bears homology to human Ube3D, which has been reported to interact with CPSF73 (Huttlin et al., 2017), suggesting that its function may be conserved.

Cft2 and Mpe1 likely have complementary roles in securing Ysh1 to the polymerase module, while Ysh1 and Mpe1 stabilize the orientation of Cft2 and Pap1 (Figure S7). The interaction of Ysh1 with Mpe1 is of critical importance, as highlighted by the lethality of a ΔUBL mutant and the deleterious effects of the Mpe1 F9S mutation (Lee and Moore, 2014). F9 lies within the UBL domain at the interface with Ysh1 (Figure 1F). The Mpe1 zinc knuckle and RING domains could be located within unassigned density in our map, possibly mediating interactions with Ysh1 or other CPF subunits (e.g., Pap1, Pta1, and Cft1) (Lee and Moore, 2014).

### Cleavage Factors Are Essential for Full Ysh1 Activation

Once Ysh1 is primed by assembly into CPF_core_, further stimulation by cleavage factors is required to achieve full nuclease activity. CF IA is a potent activator of cleavage (Figure S3B). The RRM domains of Rna15 are known to bind U- and G-rich sequences, with highest affinity for UGUUGU and UUUUUU hexamers (Pancevac et al., 2010). Consistent with photoactivable ribonucleoside-enhanced crosslinking and immunoprecipitation (PAR-CLIP) data (Baejen et al., 2014), our results suggest that CF IA binds U-rich elements downstream of the cleavage site and that removal or replacement of these downstream U-rich sequences with Cs progressively inhibits cleavage. However, we previously demonstrated that Rna14-Rna15 increases the rate of polyadenylation by the polymerase module on pre-cleaved *CYC1* substrates lacking any downstream sequences (Casañal et al., 2017). Thus, CF IA must also be able to bind upstream of the cleavage site. This is consistent with previous studies highlighting the essential role of both upstream and downstream U-rich elements for accurate 3ʹ end processing *in vivo* (Dichtl and Keller, 2001).

In contrast, CF IB alone is a poor activator of cleavage, and it enforces specificity on long substrates by suppressing aberrant secondary cleavage events in the upstream fragment (Figure S3B) (Dichtl and Keller, 2001, Minvielle-Sebastia et al., 1998). CF IB is known to bind UA repeats that comprise the efficiency element upstream of the cleavage site (Kessler et al., 1997, Pérez-Cañadillas, 2006, Valentini et al., 1999). In our assays with short substrates, only the longest (*CYC1a*) includes this element (UUUAUA; Figure 4A). CF IB can still bind to shorter RNAs (Figure 4C), which contain a UAUAUU motif proximal to the cleavage site, but it is unable to stimulate cleavage of *CYC1d.* Instead, on *CYC1d*, CF IA is sufficient, and no secondary cleavage events are observed (Figure S4E). Our short substrates lack the extensive upstream sequences that provide alternative binding sites for CF IA. This could artificially produce the observed “specificity” in the absence of CF IB.

The pre-mRNA substrate used in our assays is U rich. Both cleavage factors IA and IB bind U_15_ RNA, but CPF proteins and complexes do not (Table 3; Figure S5). Our data suggest that the polymerase module of CPF_core_ likely binds the A-rich upstream sequence (AAGAA), analogous to the AAUAAA recognition mechanism observed in recent structures of the human machinery (Clerici et al., 2017, Sun et al., 2018). This interaction would involve a surface comprising residues from Pfs2 and Yth1 (Casañal et al., 2017). CPF_core_ interaction with RNA is likely to have a fast off-rate (Figure 4C), so CF IA may be required to secure it onto the RNA.

Despite binding to both U-rich elements on the RNA and the polymerase module of CPF_core_, Rna14-Rna15 alone is unable to activate cleavage (Figure S3B). Furthermore, the Pcf11-Clp1 component of CF IA binds to *CYC1* RNA (Table 3) but does not strongly interact with CPF (Figure S6D) and cannot stimulate cleavage alone (Figure S3B). Thus, the activation mechanism cannot be explained by a model in which cleavage factors simply tether CPF to the RNA. Our results suggest that CPF and the cleavage factors do not bind in a straightforward linear manner along the RNA substrate. Instead, they may structure the RNA, binding in an intertwined, more complicated manner, explaining why the sequences that specify mRNA 3ʹ ends are degenerate. Furthermore, previous nuclear magnetic resonance (NMR) studies suggested that the Rna15 RRMs slide along RNA (Leeper et al., 2010), and this could facilitate “scanning” or repositioning of CF IA. Thus, it is conceivable that the 3ʹ end machinery is remodeled as the complex progresses from cleavage to polyadenylation and that these different states have different RNA binding modes.

### Assembly and Activation of the 3′ End Processing Machinery

**Figure 6:**
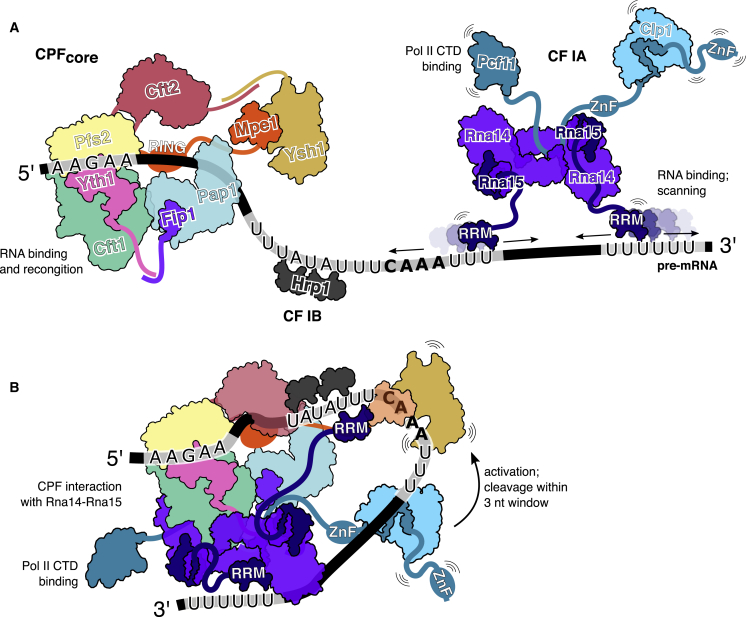
Model for 3ʹ End Formation on the Minimal *CYC1* Pre-mRNA Substrate (A) CF IA, CF IB, and CPF each preferentially bind certain RNA sequences. CF IA binds U-rich elements via interactions with Rna15 RRM domains, CF IB binds UA-rich sequences, and CPF_core_ binds the 5ʹ AAGAA element. (B) When all of the correct sequence elements are present, the 3ʹ end processing machinery can assemble into an active complex, resulting in an opening of the active site cleft of Ysh1. Pre-mRNA cleavage occurs within a 3-nt window. Some of the interactions in the model are speculative.

We propose that the CPF nuclease is only fully “licensed” to cut when the eight-subunit CPF_core_ complex and the seven proteins of CF IA and CF IB cooperatively bind multiple sequence elements on the pre-mRNA substrate in an avidity-driven mechanism (Figure 6). Once bound to the correct site on RNA, Ysh1 could be positioned close to the CAAA motif and allosterically activated by CF IA, possibly via a direct interaction between Clp1 and Ysh1 (Holbein et al., 2011). The configuration of the complex on RNA could also generate strain in the RNA backbone close to the cleavage site, facilitating cleavage at the correct position, without a requirement for strict sequence specificity. *In vivo*, RNA recognition and assembly of the 3ʹ end processing complex could also activate the Glc7 phosphatase to dephosphorylate Tyr1 of the Pol II C-terminal domain (CTD) (Schreieck et al., 2014), providing direct coupling between pre-mRNA cleavage and transcription termination.

Assembly of the 3ʹ end processing machinery may be analogous to the assembly of an active spliceosome; the active splicing complex is assembled *de novo*, on each intron, every round of splicing (Fica and Nagai, 2017). In the spliceosome, an active site is not preformed, but dynamics permit extensive remodeling of the spliceosome subunits on the RNA substrate, generating an active site and resulting in highly controlled pre-mRNA processing. Similarly, our structural model of CPF_core_ suggests that the RNA binding, cleavage, and polyadenylation activities are all adjacent to each other, but not in intimate contact. Coupling between the different enzymes of CPF may be much more dynamic than previously thought, such that RNA binding activates a series of conformational changes to open the active site of Ysh1, allowing cleavage only at the correct position (Figure 6). Both the spliceosome and the 3ʹ end processing machinery must be highly regulated to maintain the fidelity of RNA processing.

Almost all CPF subunits are conserved in humans and can be pulled down using a pre-mRNA substrate (Shi et al., 2009). The core CPSF complex appears to be composed of six subunits, CPSF160, CPSF100, CPSF73, CPSF30, hFip1, and WDR33 (Schönemann et al., 2014), which are orthologs of Cft1, Cft2, Ysh1, Yth1, Fip1, and Pfs2. The poly(A) polymerase PAP does not incorporate stably into human CPSF. It is not clear whether RBBP6, the ortholog of yeast Mpe1, is a stable component, but our docking experiments suggest that the Ysh1-Mpe1 interface is conserved in the human CPSF73 and RBBP6 proteins. This agrees with a reported role for RBBP6 in 3ʹ end processing (Di Giammartino et al., 2014). Pta1 and its human ortholog, Symplekin, are thought to play important roles in coupling 3ʹ end processing to transcription. We show that yeast Pta1 is not required for the cleavage and polyadenylation reactions themselves in our fully reconstituted system. However, Pta1 mutation disrupts CPF function in yeast extract (Zhao et al., 1999), and Symplekin is thought to be required for the cleavage activity of human CPSF (but not for polyadenylation) (Schönemann et al., 2014) and is required for histone 3ʹ end cleavage, along with CPSF73 and CPSF100 (Kolev and Steitz, 2005).

Thus, our data suggest that yeast CPF_core_ is the functional equivalent of the human CPSF-PAP complex. It is not yet clear whether the accessory cleavage factors function in the same manner in yeast and humans. Further experiments will be required to determine whether a human phosphatase module also exists and whether it assembles into an active 3ʹ end processing complex on substrate RNA.

## STAR★Methods

### Key Resources Table

**Table:** 

REAGENT or RESOURCE	SOURCE	IDENTIFIER
**Bacterial and Virus Strains**		

*E. coli* DH5α	Thermo Fisher Scientific	18258012
*E. coli* DH10 EMBacY	Geneva Biotech	
*E. coli* BL21 star pLysS	Thermo Fisher Scientific	C602003
*E. coli* TOP10	Thermo Fisher Scientific	C404010
*E. coli* PIR1	Thermo Fisher Scientific	C101010

**Chemicals, Peptides, and Recombinant Proteins**		

Insect-XPRESS protein-free insect cell medium with L-glutamine	Lonza	12-730Q
Protease Inhibitor Cocktail	Sigma-Aldrich	11836170001
Desthiobiotin	IBA	2-1000-001
Imidazole	Sigma-Aldrich	I5513
Formamide	Sigma-Aldrich	11814320001
TEMED	Sigma-Aldrich	T9281
Ammonium persulfate (APS)	Sigma-Aldrich	A3678
Accugel 19:1 acrylamide:bis-acrylamide 40% w/v mix	National Diagnostics	EC-850
Urea	VWR chemicals	28877.260
KOD Hot Start DNA Polymerase	Merck	71086
SYBR Safe DNA Gel Stain	Thermo Fisher Scientific	S33102
SYBR Green II RNA Gel Stain	Thermo Fisher Scientific	S7586
Ni-NTA Agarose	QIAGEN	30210
StrepTactin Sepharose high performance	GE Healthcare	28-9356-00
Deuterium oxide 99.9%	Millipore	1133660009
DiSuccinimidylSuberate (DSS)	Creative Molecules	001S
DiSuccinimidyl Dibutyric Urea (DSBU)	This work	
Recombinant protein: *S. cerevisiae* Cft2_-SII_	This work	N/A
Recombinant protein: *S. cerevisiae* Pap1_-SII_	This work	N/A
Recombinant protein: *S. cerevisiae*_SII-3C-_Yjr141w	This work	N/A
Recombinant protein complex: *S. cerevisiae*	This work	N/A
Ysh1(1-462)-Mpe1(1-161)_-3C-SII_		
Recombinant protein complex: *S. cerevisiae*	This work	N/A
Ysh1-Mpe1_-3C-SII_		
Recombinant protein complex: *S. cerevisiae*	This work	N/A
Ysh1-Mpe1_-3C-SII_ -Yjr141w		
Recombinant protein complex: *S. cerevisiae*	This work	N/A
Ysh1- _SII-3C-_Yjr141w		
Recombinant protein complex: *S. cerevisiae*	This work, based on Casañal et al., 2017	N/A
Cft1-Pfs2_-3C-SII_ -Yth1_-3C-8H_ -Fip1		
Recombinant protein complex: *S. cerevisiae*	This work, based on Casañal et al., 2017	N/A
“CPF_pol_” Cft1-Pfs2_-3C-SII_ -Yth1_-3C-8H_ -Fip1-Pap1		
Recombinant protein complex: *S. cerevisiae* “CPF_pol_+Cft2” Cft1-Pfs2_-3C-SII_ -Yth1-Fip1-Pap1-Cft2	This work	N/A
Recombinant protein complex: *S. cerevisiae* “CPF_core_” Cft1-Pfs2_-3C-SII_ -Yth1_-3C-8H_ -Fip1-Pap1-Cft2-Ysh1-Mpe1	This work	N/A
Endogenous protein complex: *S. cerevisiae* “CPF” Cft1-Pfs2-Yth1-Fip1-Pap1-Cft2-Ysh1-Mpe1-Pta1-Pti1-Ref2-Glc7-Ssu72	This work, based on Casañal et al., 2017	N/A
Recombinant protein: *S. cerevisiae* “CF IB” Hrp1	This work, based on Kessler et al., 1997	N/A
Recombinant protein complex: *S. cerevisiae* Rna14-Rna15	This work, based on Gordon et al., 2011	N/A
Recombinant protein complex: *S. cerevisiae* Pcf11-Clp1	This work, based on Gordon et al., 2011	N/A
Recombinant protein complex: *S. cerevisiae* “CF IA” Rna14-Rna15-Pcf11-Clp1	This work, based on Gordon et al., 2011	N/A

**Critical Commercial Assays**		

N/A		

**Deposited Data**		

Mendeley raw data (e.g., uncropped gels, MS peptides)	This work	https://doi.org/10.17632/rnsb352sx3.1
Ysh1-Mpe1 crystal structure	This work	PDB: 6I1D
Ysh1-Mpe1 cryo-EM map	This work	EMD: 0325
CPF_core_ negative stain EM map	This work	EMD: 0324
NMR structure of Rna14-Rna15 monkeytail-hinge (used for Figure 6	Moreno-Morcillo et al., 2011	PDB: 2L9B
Crystal structure of Rna14-Rna15 complex (used for Figure 6)	Paulson and Tong, 2012	PDB: 4EBA
NMR structure of C-terminal domain pf CstF-64 (used for Figure 6)	Qu et al., 2007	PDB: 2J8P
NMR structure of Hrp1-Rna15 RRMs (used for Figure 6)	Leeper et al., 2010	PDB: 2KM8
Crystal structure of Rna15 RRM with bound GU (used for Figure 6)	Pancevac et al., 2010	PDB: 2X1F
Crystal structure of Pcf11-Clp1 complex (used for Figure 6)	Dupin and Fribourg, 2014	PDB: 4C0B
Crystal structure of Pcf11-Clp1 complex (used for Figure 6)	Noble et al., 2007	PDB: 2NPI
Crystal structure of Pcf11-RNA pol II CTD complex (used for Figure 6)	Meinhart and Cramer, 2004	PDB: 1SZA
Cryo-EM structure of Cft1-Pfs2-Yth1 (used for Figures 5 and 6)	Casañal et al., 2017	PDB: 6E0J
Crystal structure of Cft2 (used for Figures 5 and 6)	Mandel et al., 2006	PDB: 2I7X
Crystal structure of Pap1-Fip1 complex (used for Figures 5 and 6)	Meinke et al., 2008	PDB: 3C66
Crystal structure of CPSF-73 (used as molecular replacement search model)	Mandel et al., 2006	PDB: 2I7T
NMR structure of Rbbp6 (used as molecular replacement search model)	Pugh et al., 2006	PDB: 2C7H

**Experimental Models: Cell Lines**		

Sf9	Oxford Expression Technologies Ltd.	600100-SF9 cells

**Experimental Models: Organisms/Strains**		

*S. cerevisiae* Ref2-TAPS (for purification of endogenous CPF):	Casañal et al., 2017	kanMX6 MATalpha pra1-1 prb1-1 prc1-1 cps1-3 ura3delta5 leu2-3 his- Parent strain JWY104

**Oligonucleotides**		

RNA and DNA sequences, with details of end-labeling	This work	See Table S2

**Recombinant DNA**		

(modified) pBig1A	This work, based on Weissmann et al., 2016	P24-63
(modified) pBig1B	This work, based on Weissmann et al., 2016	P24-64
(modified) pBig1C	This work, based on Weissmann et al., 2016	P25-1
(modified) pBig1D	This work, based on Weissmann et al., 2016	P25-2
(modified) pBig1E	This work, based on Weissmann et al., 2016	P25-6
(modified) pBig2AB	This work, based on Weissmann et al., 2016	P25-3
Pap1-SII pACEBac1 (expression). Used to make protein:	This work	P25-8
Pap1_-SII_		
Cft2-SII pACEBac1 (expression)	This work	P25-7
Used to make protein:		
Cft2_-SII_		
SII-3C-Yjr141w pIDS (assembly intermediate)	This work	P18-62
SII-3C-Yjr141w pIDS/pACEBac1 (baculovirus expression)	This work	P19-1
Used to make protein:		
_SII-3C-_Yjr141w		
Pta1_Cft2_Ysh1_Mpe1 pIDS (CPF_core_ assembly intermediate)	This work	P19-10
Cft1_Pfs2-3C-SII_Yth1-3C-8H_Pap1_Fip1 pIDC/pACEBac1 (CPF_core_ assembly intermediate and baculovirus expression) Used to make two complexes:	Casañal et al., 2017	P15-18
Cft1-Pfs2_-3C-SII_-Yth1_-3C-8H_-Fip1		
“CPF_pol_” (Cft1-Pfs2_-3C-SII_-Yth1_-3C-8H_-Fip1-Pap1)		
Cft2_Ysh1_Mpe1_Pta1_Cft1_Pfs2-3C-SII_Yth1-3C-8H_Pap1_Fip1 pIDC/pIDS/pACEBac1 (baculovirus expression). Used to make:	This work	P19-11
“CPF_core_” (Cft1-Pfs2_-3C-SII_ -Yth1_-3C-8H_ -Fip1-Pap1-Cft2-Ysh1-Mpe1)		
8H-Cft2_Ysh1_Mpe1-3C-SII pIDS (assembly intermediate)	This work	P15-13
8H-Cft2_Ysh1_Mpe1-3C-SII_Yjr141w pIDS (assembly intermediate)	This work	P18-59
SII-3C-Yjr141w_Ysh1 pIDS (assembly intermediate)	This work	P25-9
8H-Cft2_Ysh1_Mpe1-3C-SII pIDS/pACEBac1 (baculovirus expression) Used to make:	This work	P15-20
Ysh1-Mpe1_-3C-SII_		
8H-Cft2_Ysh1_Mpe1-3C-SII_Yjr141w pIDS/pACEBac1 (baculovirus expression) Used to make:	This work	P18-63
Ysh1-Mpe1_-3C-SII_ -Yjr141w		
SII-3C-Yjr141w_Ysh1 pIDS/pACEBac1 (baculovirus expression) Used to make:	This work	
Ysh1- _SII-3C-_Yjr141w		
Cft1_Pfs2-3C-SII_Yth1_Pap1_Fip1 pBig1A (assembly intermediate)	This work	P20-3
Cft2 pBig1B (assembly intermediate)	This work	P20-5
Cft2_Cft1_Pfs2-3C-SII_Yth1_Pap1_Fip1 pBig2AB (baculovirus expression) Used to make:	This work	P20-15
“CPF_pol_+Cft2” (Cft2-Cft1-Pfs2_-3C-SII_-Yth1-Fip1-Pap1)		
Cft2_Ysh1_Mpe1-3C-SII pBig1B (baculovirus expression)	This work	P20-8
Cft2_Ysh1_Mpe1-1_(1-369)_-3C-SII pBig1B (baculovirus expression)	This work	P20-29
Cft2_Ysh1_Mpe1-2_(1-270)_-3C-SII pBig1B (baculovirus expression)	This work	P20-30
Cft2_Ysh1_Mpe1-3_(1-160)_-3C-SII pBig1B (baculovirus expression)	This work	P20-31
Cft2_Ysh1_Mpe1-4_(81-441)_-3C-SII pBig1B (baculovirus expression)	This work	P20-32
Cft2_Ysh1_Mpe1-5_(161-441)_-3C-SII pBig1B (baculovirus expression)	This work	P20-33
Cft2_Ysh1-N_(1-474)__Mpe1-3C-SII pBig1B (baculovirus expression)	This work	P20-34
Cft2_Ysh1-N_(1-474)__Mpe1-1_(1-369)_-3C-SII pBig1B (baculovirus expression)	This work	P20-35
Cft2_Ysh1-N_(1-474)__Mpe1-2_(1-270)_-3C-SII pBig1B (baculovirus expression)	This work	P20-36
Cft2_Ysh1-N_(1-474)__Mpe1-3_(1-160)_-3C-SII pBig1B (baculovirus expression)	This work	P20-37
Cft2_Ysh1-N_(1-474)__Mpe1-4_(81-441)_-3C-SII pBig1B (baculovirus expression)	This work	P20-38
Cft2_Ysh1-N_(1-474)__Mpe1-5_(161-441)_-3C-SII pBig1B (baculovirus expression)	This work	P20-39
Cft2_Ysh1-C_(475-779)__Mpe1-3C-SII pBig1B (baculovirus expression)	This work	P20-40
6H-Hrp1 pOPINB (bacterial expression) Used to make:	Kessler et al., 1997	P2-43
“CF IB” (_6H-_Hrp1)		
6H-Rna14_Rna15 pETduet (bacterial expression) Used to make:	Gordon et al., 2011	P11-44
“CF IA” (_6H-_Rna14-Rna15- _6H-_Pcf11-Clp1)		
_6H-_Rna14-Rna15		
6H-Pcf11_Clp1 pSRFduet (bacterial expression) Used to make:	Gordon et al., 2011	P11-45
“CF IA” (_6H-_Rna14-Rna15- _6H-_Pcf11-Clp1)		
_6H-_Pcf11-Clp1		

**Software and Algorithms**		

DynamX 3.0	Waters	
ProteinLynx Global Server	Waters	
Stavrox	Götze et al., 2012	
msConvert	ProteoWizard	
XIA2	Winter, 2009	N/A
XDS	Kabsch, 2010	N/A
AIMLESS	Evans and Murshudov, 2013	N/A
Phaser	McCoy et al., 2007	N/A
phenix.autobuild	Terwilliger et al., 2008	N/A
COOT	Emsley et al., 2010	N/A
phenix.refine	Adams et al., 2010	N/A
MolProbity	Chen et al., 2010	N/A
ePISA, European Bioinformatics Institute, EBI	Krissinel and Henrick, 2007	N/A
PDB2PQR	Dolinsky et al., 2004	N/A
PROPKA	Li et al., 2005	N/A
APBS	Baker et al., 2001	N/A
SerialEM	Mastronarde, 2005	N/A
EPU	FEI company	N/A
MotionCor 2	Zheng et al., 2017	N/A
Gctf	Zhang, 2016	N/A
RELION 2	Scheres, 2012	N/A
EMAN	Tang et al., 2007	N/A
PyMOL 1.5.0.5	Schrödinger LLC	N/A
UCSF Chimera	Pettersen et al., 2004	N/A
HADDOCK 2.2	van Zundert et al., 2016	N/A
InkScape 0.92.3	https://inkscape.org/	N/A

**Other**		

Novex NuPAGE 4-12% Bis-Tris gels	Invitrogen	NP0323BOX
Amicon Ultra Centrifugal Filter Units	Millipore	UFC901096

NB. “SII” denotes a StrepII tag, “3C” denotes a protease cleavage site and “6H/8H” denotes a His6/His8 tag. These descriptors are positioned before or after a gene/protein name based on whether tag is N- or C-terminal

### Contact for Reagent and Resource Sharing

Further information and requests for resources should be directed to and will be fulfilled by the Lead Contact, Lori Passmore (passmore@mrc-lmb.cam.ac.uk).

### Experimental Model and Subject Details

All gene cloning, manipulation and plasmid propagation steps involving pACEBac1, pBIG1 or pBIG2 series vectors were carried out in *Escherichia coli* DH5α or TOP10 cells grown in 2 × TY or LB media supplemented with appropriate selection antibiotics. *E. coli* PIR1 cells were used for constructs in pIDC and pIDS vectors containing the R6K origin of replication. *E.coli* DH10 EmBacY cells were used for bacmid isolation.

Recombinant proteins Hrp1, Rna14–Rna15 and Pcf11–Clp1 were expressed in *E. coli* BL21 Star (DE3) cells or BL21 Star (DE3) pLysS cells grown in 2 × TY media until an OD_600nm_ of 0.6 – 1.0 was reached. Expression was induced with 1 mM IPTG for an appropriate time and temperature as described. For all other recombinant proteins and complexes, the Spodoptera frugiperda Sf9 cell line was used for baculovirus-driven overexpression. Suspension cultures were grown at 27°C, 140 rpm in Insect-XPRESS protein-free insect cell medium with L-glutamine.

Endogenous CPF was purified from *Saccharomyces cerevisiae* by using a Ref2-TAPS strain. Yeast strains were grown at 30°C in YPD media (YPD media per L: 20 g peptone, 20 g D-glucose, 10 g yeast extract) in a 120 L fermenter for 19 h. Yeast was harvested at an OD_600nm_ of 6–7.

### Method Details

#### Cloning

##### Pap1 and Cft2

Sequences encoding *S. cerevisiae* Pap1 and Cft2 were codon-optimized for *E. coli* expression and synthesized *de novo* (GeneArt). Pap1 was amplified by PCR to introduce upstream BamHI and downstream XhoI sites (primers Pap1_F and Pap1_R) prior to cloning into a modified pACEBac1 vector with an in-frame C-terminal StrepII tag (SII) and site for cleavage by 3C PreScission protease. Cft2 was also amplified and cloned as above (primers Cft2_F and Cft2_R). Constructs were confirmed by sequencing.

##### Ysh1–-Mpe1–Yjr141w, Ysh1–Mpe1 and Ysh1–Yjr141w complexes

Sequences encoding *S. cerevisiae* Ysh1, Cft2, 8H-3C-Cft2, Mpe1, Mpe1-3C-SII, Yjr141w and SII-3C-Yjr141w were codon-optimized for *E. coli* expression and synthesized with upstream BamHI and XhoI sites, and downstream KpnI and XbaI sites (GeneArt). Using the XhoI and KpnI sites, each of these genes was cloned into MultiBac vector pIDS, and multi-gene constructs were made iteratively in pIDS by using PI-SceI and BstXI digestion and ligation as described previously (Casañal et al., 2017). Final pIDS multi-gene constructs of Cft2_Ysh1_Mpe1, Cft2_Ysh1_Mpe1_Pta1, 8H-3C-Cft2_Ysh1_Mpe1-3C-SII, 8H-3C-Cft2_Ysh1_Mpe1-3C-SII_Yjr141w and SII-3C-Yjr141w_Ysh1 were then fused with empty pACEBac1 by Cre-Lox recombination to provide the Tn7L and Tn7R sites necessary for bacmid integration.

##### CPF_core_

The Cft2_Ysh1_Mpe1_Pta1 construct in pIDS was fused with the previously described (Casañal et al., 2017) Cft1_Pfs2-3C-SII_Yth1-3C-8H_Pap1_Fip1 pIDC/pACEBac1 plasmid by Cre-Lox recombination. Plasmids from > 30 colonies were then screened by restriction digest with combinations of XhoI, KpnI, PI-SceI, BstXI and I-CeuI, in order to select a clone with one copy of each gene. The resultant Cft2_Ysh1_Mpe1_Pta1_Cft1_Pfs2-3C-SII_Yth1-3C-8H_Pap1_Fip1 plasmid in pIDS/pIDC/pACEBac1 was further verified by PCR for each gene.

##### Polymerase module plus Cft2

A modified version of the biGBac system (Weissmann et al., 2016) was used. Vectors functionally equivalent to the previously-described pBIG1a,b,c,d,e and pBIG2ab,abc,abcd,abcde plasmids were created by cloning the necessary Gibson overhangs, spacers and Swa1 sites into pACEBac1. In this way, our pBIG1 series vectors were selectable using gentamycin rather than ampicillin and spectinomycin. An additional chloramphenicol resistance gene was added to our pBIG2 equivalents, so these plasmids were selectable using gentamycin and chloramphenicol.

Briefly, pACEBac1 plasmids containing Cft1, Pfs2-3C-SII and Yth1, and pIDC plasmids containing Pap1 and Fip1 were amplified by PCR using the original biGBac primers and introduced into pBIG1a by Gibson assembly. Cft2 in pIDS was amplified using modified biGBac primers that anneal to the p10 promoter and HSV-TK terminator (biGBac_pIDS_CasI_F and biGBac_pIDS_CasI_R) and was introduced into pBIG1b by Gibson assembly. Multi-gene cassettes from pBIG1a and pBIG1b were released by PmeI digestion and introduced into pBIG2ab by Gibson assembly. The final Cft1_Pfs2-3C-SII_Yth1_Fip1_Pap1_Cft2 pBIG2ab plasmid was verified by SwaI and PmeI digestion.

##### Ysh1 and Mpe1 truncations

All Ysh1 (full, Ysh1-N and Ysh1-C) and Mpe1 (full, Mpe1-1, Mpe1-2, Mpe1-3, Mpe1-4 and Mpe1-5) constructs were PCR amplified and cloned into pACEBac1 using BamHI and XhoI sites with a cleavable C-terminal StrepII tag on Mpe1. Primers are detailed in the Table S2. All clones were verified by sequencing. Ysh1 and Mpe1 truncations were then combined pairwise in all combinations for co-expression using the biGBac method (Weissmann et al., 2016). Constructs were amplified by PCR from pACEBac1 plasmids and introduced into pBIG1a by Gibson assembly, along with wild-type Cft2.

#### Recombinant baculovirus-driven protein expression

Bacmids were isolated from *E. coli* DH10 EmBacY cells, as described (Bieniossek et al., 2008). Each bacmid was verified by PCR for the genes of interest. To make P1 virus, 6-well dishes were seeded with 1.0 × 10^6^ *Sf9* cells per well in 2.0 mL InsectExpress medium (Lonza). Cells were transfected with 10 μg bacmid per well, using FugeneHD reagent as described by the manufacturer (Promega). Four days post-transfection, cells were checked for fluorescence, conditioned medium was harvested, diluted 1:1 with fresh medium containing 20% FBS and 0.2 μm-filtered. P1 virus was stored at 4°C in the dark. P2 (amplified) virus was prepared by infecting suspension cultures of *Sf9* cells at 2.0 × 10^6^/mL with 1% v/v P1 virus and incubating for 3–4 days (140 rpm, 27°C). Cells were checked for fluorescence, pelleted by centrifugation (1000 × g, 5 min) and supernatant was 0.2 μm-filtered. Large-scale expression cultures were then set up by infecting 4–12 L suspension cultures of *Sf9* cells at 2.0 × 10^6^/mL with 1% v/v P2 virus. Following incubation (140 rpm, 27°C), cells were harvested by centrifugation (1000 × g, 10 min, 4°C) 48 hours post-infection, washed in ice-cold PBS and snap frozen in liquid nitrogen. Pellets were stored at −80°C.

#### Protein purification

CF IA, CF IB, Rna14–Rna15 and Pcf11–Clp1 were expressed in *E. coli* and purified as described previously (Gordon et al., 2011, Kessler et al., 1997). Polymerase module complexes were expressed in *Sf9* cells and purified as described previously (Casañal et al., 2017). Endogenous CPF was purified from a yeast strain where the *REF2* gene was modified to contain a TAPS tag as described previously (Casañal et al., 2017).

For all other complexes, a standardized protocol was followed. Cell pellets from 2 L *Sf9* cells were resuspended in 200 mL 50 mM HEPES pH 7.9, 150 mM NaCl, 0.5 mM Mg(OAc)_2_, 1 mM TCEP supplemented with 50 μg/mL RNase, 50 μg/mL DNase and EDTA-free protease inhibitors, and lysed by sonication. Lysate was cleared by centrifugation (39,000 × g, 45 min, 4°C) prior to incubation (2–4 h, 4°C,) with 2 mL of StrepTactin Sepharose HP resin (GE Healthcare) pre-equilibrated in the same buffer. Beads were washed in batch four times with 200 mL buffer (as above, but without DNase, RNase or protease inhibitors) by centrifugation (600 × g, 10 min, 4°C) and re-suspension. Washed beads were pooled to a gravity column, then protein was eluted with buffer supplemented with 6 mM desthiobiotin. If cleavage of the Strep tag was required, 1 mg PreScission protease was added and the mixture incubated (4–12 h, 4°C). The eluate was then diluted with 50 mM HEPES pH 7.9 to reduce the salt concentration to 100 mM NaCl before anion exchange chromatography. Samples were loaded onto a 1 mL Mono Q 5/50 GL column (GE Healthcare) and eluted over a 100 CV gradient from 100–1000 mM NaCl. This separation allowed the removal of contaminants, excess subunits, PreScission protease and degraded complexes. Fractions containing proteins/complexes of interest were then pooled and concentrated using an Amicon® Ultra centrifugal filter unit with an appropriate molecular weight cut-off (100K for CPF_core_ and polymerase module; 50K for nuclease sub-complexes; 30K for Pap1 and Cft2). Size exclusion chromatography was then performed using a Superose 6 Increase column (either 3.2/300, 10/300 or 16/600 depending on yield) pre-equilibrated in 10 mM HEPES pH 7.9, 150 mM NaCl, 1.0 mM TCEP. Purified proteins/complexes were either used immediately for making cryo-EM grids or growing crystals, or were concentrated as above (10–20 μM), flash-frozen in liquid nitrogen and stored at −80°C.

#### Cleavage and Polyadenylation activity assays

##### Long CYC1 substrate

RNA sequences are described in Table S2. Unless otherwise stated, cleavage reactions of 20 μL comprised 100 nM unlabeled 259 nt *CYC1* substrate, 100 nM enzyme (e.g., CPF_core_, Ysh1–Mpe1), 200 nM CF IA and 200 nM CF IB in a buffer of 10 mM HEPES pH 7.9, 125 mM NaCl, 2.0 mM Mg(OAc)_2_, 1.0 mM DTT and 1 U/μL RiboLock (Thermo). For experiments to test polyadenylation, 2.5 mM ATP was also included in the buffer. Reactions were started by mixing 10 μL 200 nM RNA with 10 μL 200 nM protein, 400 nM CF IA and 400 nM CF IB, and incubated at 30°C in a thermal cycler. After 10, 30 and 90 min, reactions were stopped by the addition of 20 μL stopping buffer (80% v/v formamide, 1 M NaCl, 0.05% w/v bromophenol blue) and heating (72°C, 5 min). Negative control reactions were also set up containing only the cleavage factors and RNA for the duration of the longest time point. Samples were then analyzed by denaturing 6% acrylamide/7 M Urea PAGE (TBE, 20W, 25 min). Gels were stained in TBE with 1/10,000 SyBr Green (Life Technologies) for 15 min at room temperature, de-stained for 20 min in distilled water and imaged with a ChemiDoc XRS+ (BioRad).

##### Short substrates

RNA sequences are described in Table S2. Reactions were set up as above, but using fluorescent RNA oligonucleotide substrates labeled at the 5′ end with FAM and at the 3′ end with AlexaFluor 647 (IDT). After stopping the reaction, reactions with substrates ≥ 36 nt were analyzed by denaturing 20% acrylamide/7 M Urea PAGE and reactions with substrates ≤ 30 nt were analyzed by denaturing 25% acrylamide/6 M Urea PAGE (TBE, 20W, 30 min). Gels were then scanned twice with a Typhoon FLA-7000 (GE) using the 473 nm laser/Y520 filter to detect FAM and the 635 nm laser/R670 filter to detect A647. Greyscale images from each channel were contrast-normalized to the same background intensity and then layered as a false-color TIF file (GIMP).

#### Pull-down experiments

Strep-tagged ‘bait’ proteins and complexes in pull-down buffer (10 mM HEPES pH 7.9, 150 mM NaCl, 0.5 mM Mg(OAc)_2_, 0.05% Tween-20) were first immobilized on equilibrated Streptactin (GE) beads. 100 μL of bait protein at 1.5 μM was added to 40 μL beads in 860 μL pull-down buffer and incubated for 60 min at 4°C. Beads were then washed twice in 1.0 mL pull-down buffer (600 × g, 5 min, 4°C) and divided equally between four tubes, each containing 10 μL bait-loaded beads and 250 μL pull-down buffer. Unloaded beads were also included as negative controls. 20 μL of untagged ‘prey’ proteins CF IA, CF IB, Rna14–Rna15 and Pcf11–Clp1 at 4.0 μM concentration were added to each bait and allowed to bind for 60 min at 4°C. Beads were then washed four times in 1.0 mL pull-down buffer (600 × g, 5 min, 4°C) prior to elution by addition of SDS-PAGE loading buffer (50 mM Tris-HCl pH 6.8, 10% v/v glycerol, 2% w/v SDS, 0.05% w/v bromophenol blue), heating (95°C, 2 min), and analysis by Bis-Tris 4%–12% gradient SDS-PAGE in MOPS-SDS buffer (200 V, 50 min). Bands were visualized by staining with InstantBlue (Expedeon).

#### Electrophoretic mobility shift assay (EMSA)

5′-FAM fluorescently-labeled RNA oligonucleotides (IDT) were dissolved in DEPC water. For each sequence tested for binding, a series of reactions were prepared on ice, each containing 1.0 μL 500 nM RNA, 1.0 μL 10 × loading dye (0.4% w/v orange G, 50% v/v glycerol, 1 mM EDTA) and 8.0 μL of serially-diluted protein at concentrations of 5.0, 2.5, 1.25, 0.62 and 0.31 μM in 10 mM HEPES pH 7.9, 150 mM NaCl and 0.5 mM Mg(OAc)_2_. This gave final binding reactions of 10 μL with 50 nM RNA, 1 × loading dye and proteins at concentrations of 4.0, 2.0, 1.0, 0.5 and 0.25 μM. Samples were incubated on ice for 30 min prior to analysis by native 6% acrylamide/TBE PAGE (40 min, 100 V constant). Gels were then scanned with a Typhoon FLA-7000 (GE) using the 473 nm laser/Y520 filter.

#### Chemical synthesis of BuUrBu

All starting materials were purchased from Sigma Aldrich unless otherwise stated and used without any further purification. NMR spectra were acquired on a *Bruker* Avance-III operating at 400 MHz, using deuterated solvents as detailed and at ambient temperature (300K). Notation for the ^1^HNMR spectral splitting patterns includes: singlet (*s*), triplet (*t*), quintet (*quint*) and broad singlet (*bs*). Chemical shifts (δ) are quoted in ppm and coupling constants (*J*) are quoted in Hertz. ^1^HNMR spectra are reported using the residual non deuterated solvent as internal standard ((CD_3_)_2_CO ^1^H, 2.05 ppm, CD_3_OD ^1^H, 3.31 ppm, (CD_3_)_2_SO ^1^H, 2.50 ppm).

##### Synthesis of 2,5-dioxopyrrolidin-1-yl 2,2,2-trifluoroacetate (**NHS-TFA, 2**)

**Figure:**
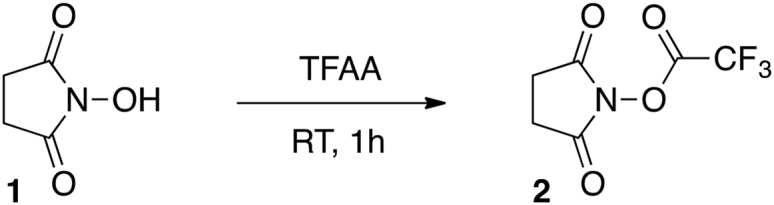

The synthesis was performed according to a previously published method (Adamczyk et al., 2000). Trifluoroacetic anhydride (TFAA, 9.2 mL, 66.2 mmol) was cooled to 0°C and NHS (**1**, 5.0 g, 43.4 mmol) added in one portion under N_2_. The reaction was warmed to RT and stirred for 1 h. Excess of TFAA/TFA was removed under reduced pressure. The solid residue was then dissolved in toluene (25 ml) and the remaining amounts of TFAA/TFA were co-evaporated on a rotary evaporator. The process was repeated twice with toluene and three times with dichloromethane (DCM, 3 × 25 ml), providing **2** as a white solid. Yield: quantitative.

^1^H NMR (400 MHz, (CD_3_)_2_CO) δ 3.02 (4H, *s*).

##### Synthesis of 4,4’-(carbonylbis(azanediyl))dibutyric acid (**AcBuUrBuAc, 4**)

**Figure:**
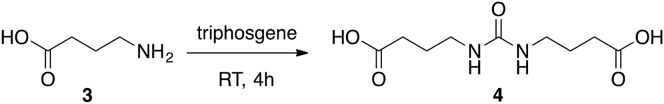

The synthesis was performed with a modified version of previously published methods. (Müller et al., 2010, Zhao et al., 2002). γ-Aminobutyric acid (**3**, 2.27 g, 22.0 mmol) and K_2_CO_3_ (3.59 g, 26.0 mmol) were dissolved in 10 mL of H_2_O in a three-necked round bottom flask and the solution was cooled to 0°C. Simultaneously, a solution of triphosgene (683 mg, 2.3 mmol) in toluene (2 ml) and a solution of K_2_CO_3_ (4.0 g, 29.0 mmol) in H_2_O (10 mL) were added dropwise (over 15 min) to the vigorously stirred reaction mixture. The reaction was brought to RT and additionally stirred for 4 h. At the end of the reaction, the toluene layer was discarded and the aqueous layer was extracted twice with diethyl ether. The aqueous solution was then acidified with concentrated HCl (final pH ∼2) and **4** precipitated as a crystalline white solid upon cooling. The precipitate was filtered and washed with a small portion of ice-cold water. Yield: 22%

^1^H NMR (400 MHz, CD_3_OD) δ 3.15 (4H, *t*, *J* = 8 Hz), 2.32 (4H, *t*, *J* = 8 Hz), 1.76 (4H, *quint*, *J* = 8 Hz).

##### Synthesis of bis(2,5-dioxopyrrolidin-1-yl) 4,4’-(carbonylbis(azanediyl))dibutyrate (**BuUrBu,5**)

**Figure:**
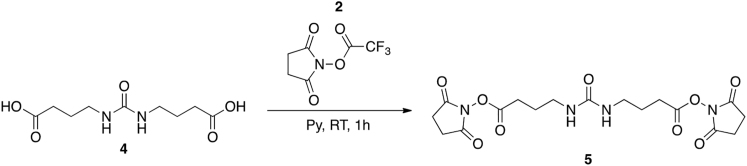

The synthesis was performed according to previously published methods (Müller et al., 2010, Rao et al., 2002). AcBuUrBuAc (**4**, 100 mg, 0.43 mmol) was dissolved in dry pyridine (2 mL) under N_2_ and cooled to 0°C. NHS-TFA (**2**, 546 mg, 2.6 mmol) was added in one portion under a flow of N_2_ and the reaction was brought to RT over 2 h. After addition of ethyl acetate the raw product was isolated by filtration and then suspended in a DCM:MeOH mixture. The insoluble components were removed by filtration and the filtrate was dried on a rotary evaporator. Compound **5** was isolated as a white solid. Yield: 83%

^1^H NMR (400 MHz, (CD_3_)_2_SO) δ 5.97 (2H, *bs*), 3.05 (4H, *t*, *J* = 8 Hz), 2.81 (8H, *s*), 2.65 (4H, *t*, *J* = 8 Hz), 1.72 (4H, *quint*, *J* = 8 Hz).

#### Cross-linking coupled to mass spectrometry

The purified complexes Ysh1–Mpe1, Ysh1–Yjr141w and Ysh1–Mpe1–Yjr141w were cross-linked with the *N*-hydroxysuccinimide (NHS) esters disuccinimidyl dibutyric urea (BuUrBu also known as DSBU) and the isotopically-coded disuccidinimidyl suberate (DSS H_12_/D_12_) purchased from Creative Molecules (Canada). The cross-linking reactions were incubated for 45 min at 37°C at a final excess of either 100- or 50-fold that of the protein concentration. The reactions were quenched by adding NH_4_HCO_3_ to a final concentration of 50 mM and incubating for further 15 min.

The cross-linked samples were freeze-dried and resuspended in 50 mM NH_4_HCO_3_ to a final protein concentration of 1 mg/mL, reduced with 10 mM DTT and alkylated with 50 mM iodoacetamide. Following alkylation, proteins were digested with trypsin (Promega, UK) at an enzyme-to-substrate ratio of 1:20, overnight at 37°C. The samples were acidified with formic acid to a final concentration of 2% (v/v) and the peptides fractionated by peptide size exclusion chromatography, using a Superdex Peptide 3.2/300 column (GE Healthcare) with 30% (v/v) acetonitrile/0.1% (v/v) TFA as mobile phase and at a flow rate of 50 μL/min. Fractions were collected every 2 min from 1.0 –1.7 mL elution volume, lyophilized and resuspended in 2% (v/v) acetonitrile and 2% (v/v) formic acid.

The fractions were analyzed by nano-scale capillary LC–MS/MS using an Ultimate U3000 HPLC (ThermoScientific Dionex, USA) to deliver a flow of approximately 300 nL/min. A C18 Acclaim PepMap100 5 μm, 100 μm × 20 mm nanoViper (ThermoScientific Dionex, USA), trapped the peptides before separation on a C18 Acclaim PepMap100 3 μm, 75 μm × 250 mm nanoViper (ThermoScientific Dionex, USA). Peptides were eluted with a gradient of acetonitrile. The analytical column outlet was directly interfaced via a nanoflow electrospray ionization source, with a hybrid dual pressure linear ion trap mass spectrometer (Orbitrap Velos, ThermoScientific, USA). MS data were acquired in data-dependent mode. High-resolution full scans (R = 30,000, m/z 300-2000) were recorded in the Orbitrap. For samples cross-linked with DSS, MS/MS scans of the 20 most intense MS peaks were recorded in the linear quadrupole ion trap (LTQ) after CID activation (collision energy 35). For samples cross-linked with the CID cleavable BuUrBu, the 3 most intense MS peaks were CID activated (collision energy 30) and high resolution MS^2^ spectra were acquired (R = 30,000, m/z 300-2000). After *in-source* CID activation (collision energy 30), the three most intense ions recoded in MS^2^ spectra were CID activated (collision energy 35) and the MS^3^ spectra were recorded in the LTQ ion trap.

For data analysis, Xcalibur raw files were converted into the MGF format through MSConvert (Proteowizard; (Kessner et al., 2008)) and used directly as input files for StavroX1 (Götze et al., 2012) and MeroX (Götze et al., 2015). Searches were performed against an *ad hoc* protein database containing the sequences of the complexes and a set of randomized decoy sequences generated by the software. The following parameters were set for the searches: maximum number of missed cleavages 3; targeted residues K, S, Y and T; minimum peptide length 5 amino acids; variable modifications: carbamidomethyl-Cys (mass shift 57.02146 Da), Met-oxidation (mass shift 15.99491 Da); DSS cross-links mass shift 138.06808 Da (precision: 10 ppm MS^1^ and 0.8 Da MS^2^), BuUrBu modification fragments: 85.05276 Da and 111.03203 (precision: 5 ppm MS^1^ and 10 ppm MS^2^); False Discovery Rate cut-off: 5%. Finally, each fragmentation spectrum was manually inspected and validated.

#### Hydrogen-deuterium exchange mass spectrometry (HDX-MS)

Deuterium exchange reactions of three complexes, Ysh1–Mpe1, Ysh1–Yjr141w and Ysh1–Mpe1–YJR141W were initiated by diluting the protein in D_2_O (99.8% D_2_O ACROS, Sigma, UK) in 10 mM HEPES pH 7.9, 150 mM NaCl, 1mM TCEP to give a final D_2_O percentage of ∼95%. For all experiments, deuterium labeling was carried out at 23°C (unless otherwise stated) at four points, 0.3 s (3 s on ice), 3 s, 30 s and 300 s in triplicate. The labeling reaction was quenched by the addition of chilled 2.4% v/v formic acid in 2 M guanidinium hydrochloride and immediately frozen in liquid nitrogen. Samples were stored at −80°C prior to analysis.

The quenched protein samples were rapidly thawed and subjected to proteolytic cleavage by pepsin followed by reversed phase HPLC separation. Briefly, the protein was passed through an Enzymate BEH immobilized pepsin column, 2.1 × 30 mm, 5 μm (Waters, UK) at 200 μl/min for 2 min, the peptic peptides were trapped and desalted on a 2.1 × 5 mm C18 trap column (Acquity BEH C18 Van-guard pre-column, 1.7 μm, Waters, UK). Trapped peptides were subsequently eluted over 11 min using a 3%–43% gradient of acetonitrile in 0.1% v/v formic acid at 40 μL/min. Peptides were separated on a reverse phase column (Acquity UPLC BEH C18 column 1.7 μm, 100 mm x 1 mm, Waters, UK) and detected on a SYNAPT G2-Si HDMS mass spectrometer (Waters, UK) over a *m/z* of 300–2000, with the standard electrospray ionization (ESI) source with lock mass calibration using [Glu1]-fibrino peptide B (50 fmol/μL). The mass spectrometer was operated at a source temperature of 80°C and a spray voltage of 2.6 kV. Spectra were collected in positive ion mode.

Peptide identification was performed by MS^e^ (Silva et al., 2005) using an identical gradient of increasing acetonitrile in 0.1% v/v formic acid over 11 min. The resulting MS^e^ data were analyzed using Protein Lynx Global Server software (Waters, UK) with an MS tolerance of 5 ppm.

Mass analysis of the peptide centroids was performed using DynamX software (Waters, UK). Only peptides with a score > 6.4 were considered. The first round of analysis and identification was performed automatically by the DynamX software, however, all peptides (deuterated and non-deuterated) were manually verified at every time point for the correct charge state, presence of overlapping peptides, and correct retention time. Deuterium incorporation was not corrected for back-exchange and represents relative, rather than absolute changes in deuterium levels. Changes in H/D amide exchange in any peptide may be due to a single amide or a number of amides within that peptide.

#### Protein complex crystallization

Purified Ysh1–Mpe1 complex was concentrated to 8.3 mg/ml in 10 mM HEPES pH 7.9, 150 mM NaCl, 1.0 mM TCEP. Crystals were grown at room temperature by sitting drop vapor diffusion against an 80 μL reservoir of 26% w/v PEG 3000, 0.1 M CHES pH 8.7. The final drop of 400 nL comprised 200 nL protein and 200 nL crystallization buffer. Crystals were cryo-protected by the addition of 0.5 μL crystallization buffer supplemented with 20% v/v glycerol, prior to harvesting in nylon loops and flash-cooling by plunging into liquid nitrogen.

#### X-ray data collection, structure determination, refinement and analysis

Diffraction datasets (Table 1) of 900 images were recorded at Diamond Light Source, beamline I04-1 on a Pilatus 6M detector (Dectris), using an oscillation range of 0.2° and an exposure time of 0.2 s per image. Data were collected at a temperature of 100 K. Data were processed with the XIA2 (Winter, 2009) automated pipeline, using XDS (Kabsch, 2010) for indexing and integration, and AIMLESS (Evans and Murshudov, 2013) for scaling and merging. Resolution cut-off was decided by a CC_1/2_ value > 0.5 in the highest resolution shell. The structure was solved by two-component molecular replacement with Phaser (McCoy et al., 2007), using the crystal structure of human CPSF73 (PDB: 2I7V; (Mandel et al., 2006)) and the NMR structure of human RBBP6 (PDB: 2C7H; (Pugh et al., 2006)) as sequential search models. Following rigid-body refinement of the molecular replacement solution, phenix.autobuild (Terwilliger et al., 2008) was successful in placing 68% of residues. The model was completed manually by iterative cycles of model-building using COOT (Emsley et al., 2010) and refinement with phenix.refine (Adams et al., 2010). Evaluation by MolProbity was used throughout the process to preserve correct model geometry. The calculation of buried surface area was carried out using the ePISA service at the European Bioinformatics Institute, EBI. For the electrostatic potential calculations, partial charges were first assigned using PDB2PQR (Dolinsky et al., 2004), implementing PROPKA (Li et al., 2005) to estimate protein pKa values. Electrostatic surfaces were then calculated using APBS (Baker et al., 2001).

#### Protein cross-linking for electron microscopy

CPF_core_ at a concentration of 3.0 mg/mL (5.4 μM) in 50 mM HEPES pH 7.9, 150 mM NaCl, 1 mM TCEP and 1 mM Mg(OAc)_2_ was cross-linked on ice for 20 min by the addition of BS3 to final concentration of 1.0 mM. The reaction was then quenched by the addition of 1 M NH_3_HCO_3_ pH 8.0, and crosslinking was confirmed by 3%–7% gradient Tris-acetate PAGE. Cross-linked complexes were then separated from aggregates by size-exclusion chromatography (Superose 6 increase 3.2/300) in a buffer containing 10 mM HEPES pH 7.9, 150 mM NaCl, 0.5 mM Mg(OAc)_2_, 1 mM TCEP. Fractions that eluted at the same volume as non-cross-linked material were pooled.

#### Electron cryo-microscopy (cryo-EM)

##### Ysh1–Mpe1–Yjr141w

Cross-linked Ysh1–Mpe1–Yjr141w was diluted to 350 nM in 10 mM HEPES, pH 7.9, 150 mM NaCl. Cryo samples were then prepared on UltraAuFoil R1.2/1.3 gold supports (Russo and Passmore, 2014). Grids were made hydrophilic by plasma treatment with 9:1 argon:oxygen for 30 s. Three microliters of sample was applied to grids, blotted for 10 s, and vitrified by plunging into liquid ethane using a Vitrobot MK IV (FEI) at 4°C, 100% relative humidity. Preliminary micrographs suggested that the complex was substantially smaller than expected (∼8 nm). Thus, to enhance contrast we used a Volta phase plate with applied defocus and collected data only in holes with thin ice, on specimens prepared on all-gold supports. Micrographs were collected at IGBMC, Strasbourg on a C_s_-corrected FEI Titan Krios microscope (FEI) operating at 300 keV and equipped with a Volta phase plate, K2 camera (Gatan) and Gatan Image Filter (GIF) with a slit-width of 20 eV. At 105,000 × magnification, the calibrated pixel size was 1.09 Å. Gain-normalized, LZW-compressed TIF movies with a total electron dose of ∼45 e^-^/Å^2^ were recorded in super-resolution mode over 9 s (42 frames) with applied defocus of −0.5 μm. SerialEM software was used for automatic acquisition (Mastronarde, 2005). After manual inspection, 994 micrographs were used in subsequent image processing.

Movie frames were aligned and a dose-weighted average calculated with MotionCor2 (Zheng et al., 2017). The contrast transfer function (CTF) and image phase shift was estimated using Gctf (Zhang, 2016) All subsequent image-processing steps were carried out in RELION2 (Scheres, 2012). Initially, auto-picking of 509,298 particles was carried out using a Gaussian blob as a reference. After several rounds of 2D and 3D classification (necessary to both clean dataset and enrich rare views), four classes that represented different views were low-pass filtered to 20 Å and used for template-based auto-picking. The resultant 429,703 particles were better centered, and subsequent 2D and 3D classification led to a subset of 43,308 particles that contributed to the final map with an anisotropic resolution of 4.8 Å in the best direction. The resolution estimation reported is based on the gold standard Fourier shell correlation (FSC) at 0.143, and the calculated FSC is derived from comparisons between reconstructions from two independently refined half-sets. The map was post-processed, and the final reconstruction was filtered to 6 Å.

##### CPF_core_

Cross-linked CPF_core_ was diluted to 250 nM in 10 mM HEPES, pH 7.9, 150 mM NaCl. Cryo samples were prepared as above, but using a 6 s blot time. Test datasets were also acquired on Quantifoil R1.2/1.3 grids coated with graphene oxide or amorphous carbon, but this did not improve the specimen. Micrographs were collected at MRC-LMB on a Tecnai G2 Polara microscope (FEI) operating at 300 keV, using a Falcon III camera (FEI). At 59,000 × magnification, the calibrated pixel size was 1.78 Å. Uncompressed movies were acquired in integration mode with a total electron dose of ∼60 e^-^/Å^2^ over 2 s (62 frames) with applied defocus of −2.5, −3.0, −3.5, −4.0 and −4.5 μm. After manual inspection, 704 micrographs were used in subsequent image processing.

Movie frames were aligned and averaged with MotionCorr. The contrast transfer function (CTF) was calculated using Gctf. All further image-processing steps were performed in RELION2. Initially, a subset of 20 micrographs with different defocus values were used to manually pick ∼5,000 particles for initial reference-free 2D classification. The resulting 2D classes were low-pass filtered to 20 Å and used as templates for automated particle-picking. From the initial 506,293 particles picked, iterative 2D classification was used to clean the dataset. In the cleaned data, 116,800 particles formed classes in which only density for polymerase module was visible; this was confirmed by a map at ∼8 Å resolution following 3D refinement. The remaining 3973 particles formed classes in which additional globular subunits were visible.

#### Negative stain electron microscopy

Cross-linked CPF_core_ was diluted to 35 nM in 10 mM HEPES pH 7.9, 150 mM NaCl, 0.5 mM Mg(OAc)_2_, 1 mM TCEP. Copper grids (400-mesh) with continuous thin carbon film were made hydrophilic by glow-discharge in air for 20 s. Three microliters of sample was applied to the support and allowed to adsorb for 60 s before wicking away with filter paper. Grids were then applied sequentially to two 30 μl drops of 2% w/v uranyl acetate, first to wash (quick) and then to stain (30 s). Excess stain was then wicked away with filter paper until dry. Micrographs were acquired on a Tecnai Spirit microscope (FEI) operating at 120 keV, equipped with an Ultrascan 1000 CCD camera (Gatan). At 26,000 × magnification, the calibrated pixel size was 3.98 Å. 618 micrographs were acquired in regions of equivalent stain thickness at −0.6 μm defocus with a total electron dose of 40–60 e^-^/Å^2^ over 2 s.

274,806 particles were initially picked with e2boxer (Tang et al., 2007). All subsequent processing was performed in RELION2. Several rounds of reference-free 2D classification were used clean bad particles from the dataset, and to discard particles classified on the basis of stain thickness, leaving a final subset of 107,817 particles in equivalently thick stain. A common lines approach was used to generate an initial model, which was refined to give a final map with a resolution of 20 Å. One round of 3D classification was then used to separate conformational heterogeneity, prior to a final 3D refinement to yield a map with a resolution of 25 Å from 23,969 particles. The resolution estimation is based on the gold standard Fourier shell correlation (FSC) at 0.143, and the calculated FSC is derived from comparisons between reconstructions from two independently refined half-sets.

#### Visualization of structural data

All structural figures depicting crystallographic data (cartoon, stick and surface representations) were rendered in PyMOL (Schrödinger LLC). Structural figures of EM maps with docked components were rendered in Chimera. For the model in Figure 6, to-scale cleavage factor protein outlines were prepared in Inkscape based on PyMOL-rendered surface representations of PDB:s 2L9B (Moreno-Morcillo et al., 2011), 4EBA (Paulson and Tong, 2012), 2J8P (Qu et al., 2007), 2KM8 (Leeper et al., 2010), 2X1F (Pancevac et al., 2010), 4C0B (Dupin and Fribourg, 2014), 2NPI (Noble et al., 2007) and 1SZA (Meinhart and Cramer, 2004).

#### Docking

To prepare the CPSF73 and RBBP6 protein models for docking (PDB: 2I7V and 2C7H, respectively), all solvent atoms and ligands were removed. Docking was performed with HADDOCK 2.2 (van Zundert et al., 2016) using the WeNMR grid service (Wassenaar et al., 2012). For preparation of ambiguous interactions restraints, CPSF73 residues M50, D51, Y55, D57 and RBBP6 residues F13, K45, R78, R79 and P81 were specified as active. For both molecules, passive residues were defined automatically within a 6.5 Å radius of active residues.

### Quantification and Statistical Analysis

Crystallographic calculations (e.g., integration, scaling, merging) were performed as described in methods text, using the default software parameters unless otherwise stated. Processing and refinement statistics are detailed in Table 1.

#### Statistical evaluation of HDX-MS data in DynamX 3.0

The HDX-MS data collected in triplicate in this study allowed us to calculate the corresponding uncertainty for each difference in deuterium uptake data point expressed as one standard deviation (SD), as described previously (Houde et al., 2011). The average of all the individual experimentally determined SD values for all calculated mean difference data points for each peptide at all charge states and replicates was determined and the value used as the best estimate of SD for any difference point. This value was then used to calculate the standard error of the mean (SEM) for any mean difference value, as obtained from the average of the three separate HDX-MS experiments conducted on the same sample. Using this value for SEM and multiplying it by the appropriate Student’s t table value for the 98% confidence gave an estimated 98% confidence limit of ∼0.5 Da for any mean value for difference calculated from three replicate HDX-MS experiments. This value is represented as the gray dashed lines on the difference plots. Any value outside of these limits can be considered significant.

#### Scoring cross-linked peptides in Stavrox 3.6.6

The scoring algorithm used by Stavrox reflects the quality of the respective fragment ion mass spectrum, which is calculated from the number of signals above a specified signal-to-noise ratio. The score is based on the number of identified b- and y-type ions as well as on the number and length of the ion series (Götze et al., 2012). To estimate the quality of a fragment ion spectrum the total number of fragment ions above the threshold as well as the number of signals with relative intensities above 10% are taken into account. The length of the respective b- or y-type ion series also influences the score. Each b- and y-type ion series of every crosslinked peptide is divided by the total length of the peptide. A logarithmic conversion of this probability yields the score that is displayed by StavroX.

#### Decoy analysis

False-positive peptide identifications and hence false discovery rate (FDR) were determined by searching the acquired LS-MS/MS data against a decoy database, generated by inverting the true sequences supplied in the fasta data file. This inversion can only lead to false-positives. An FDR of 5% was applied to this dataset.

### Data and Software Availability

The accession number for the atomic coordinates and structure factors for the Ysh1–Mpe1 X-ray crystal structure reported in this paper is PDB: 6I1D. The accession numbers for the CPF_core_ negative-stain EM map and the Ysh1–Mpe1 cryo-EM map reported in this paper are EMDB: 0324 and EMDB: 0325, respectively. Raw data (e.g., uncropped, unannotated gels, plots, lists of MS peptides) corresponding to individual figure panels have been deposited in Mendeley Data (https://doi.org/10.17632/rnsb352sx3.1).

## Supplemental Information

Document S1. Figures S1–S7, Tables S1 and S2, and PDB Validation Report

Document S2. Article plus Supplemental Information
