# Supplementary material for: Activation of the Endonuclease that Defines mRNA 3′ Ends Requires Incorporation into an 8-Subunit Core Cleavage and Polyadenylation Factor Complex
Source: Mol Cell. 2019 Mar 21;73(6):1217–1231.e11. doi: 10.1016/j.molcel.2018.12.023 (PMC6436931; doi:10.1016/j.molcel.2018.12.023)
Supplement: Document S1. Figures S1–S7, Tables S1 and S2, and PDB Validation Report [file mmc1.pdf]

Molecular Cell, Volume *73*

Supplemental Information

Activation of the Endonuclease that Defines mRNA 3′ Ends Requires Incorporation into an 8-Subunit Core Cleavage and Polyadenylation Factor Complex

Chris H. Hill, Vytautė Boreikaitė, Ananthanarayanan Kumar, Ana Casañal, Peter Kubík, Gianluca Degliesposti, Sarah Maslen, Angelica Mariani, Ottilie von Loeffelholz, Mathias Girbig, Mark Skehel, and Lori A. Passmore

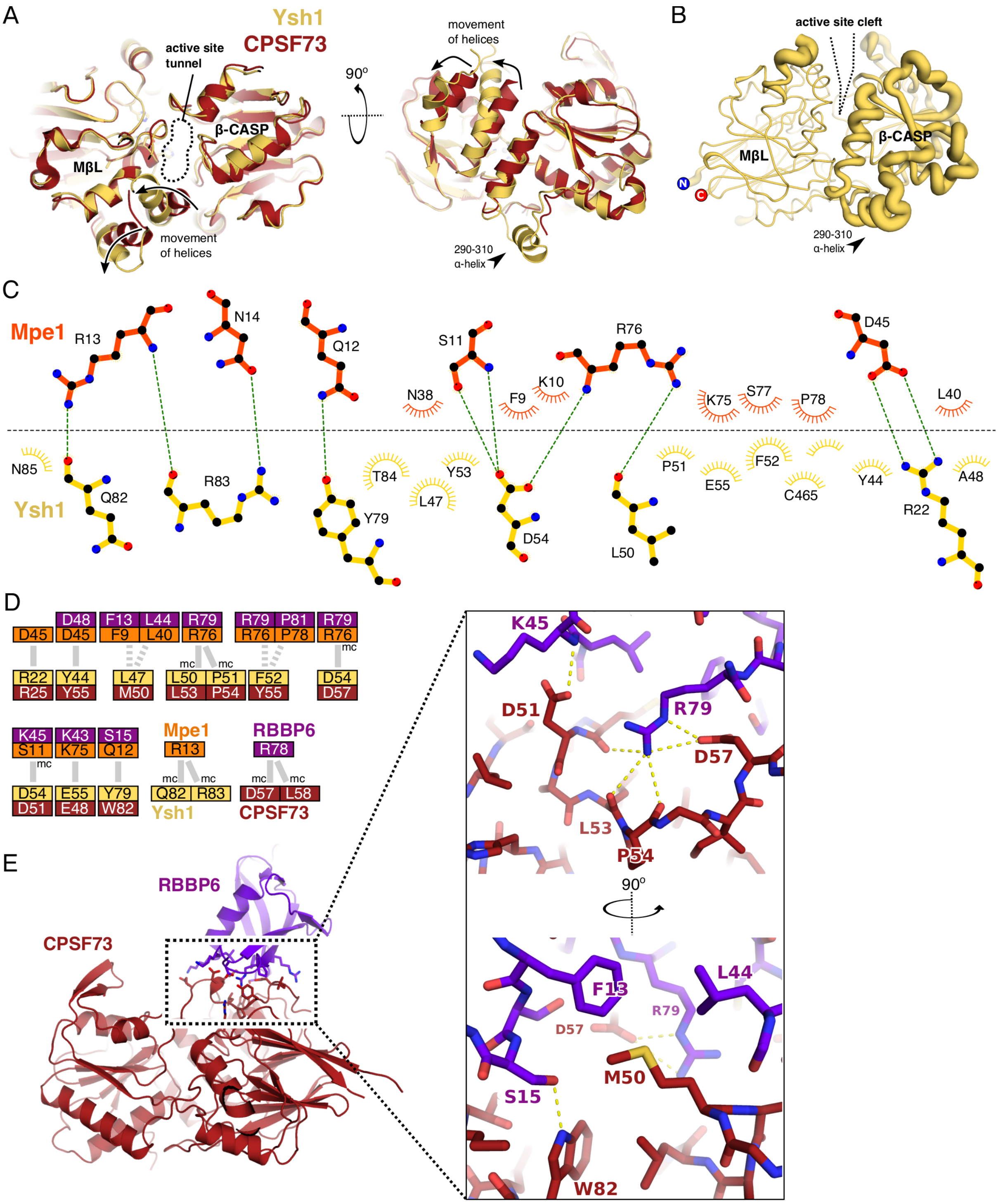

Figure S1. Details of the Ysh1–Mpe1 X-ray crystal structure. Related to Figure 1.
(**A**) Secondary structure-based alignment of yeast Ysh1 (yellow; this study) with human CPSF73 (red; Mandel et al., 2006). Two orthogonal views are shown. The structure of yeast Ysh1 is similar to CPSF73 (RMSD = 0.73 Å over 2390 atoms) and yeast Cft2 (RMSD = 3.65 Å over 1735 atoms). (**B**) Cartoon representation of the Ysh1 N-terminal catalytic domain, in which ribbon thickness is proportional to atomic *B* factor. (**C**) Summary of the intermolecular interactions at the Ysh1-Mpe1 interface. Hydrogen bonds and electrostatic interactions are represented by green dashed lines. Hydrophobic interactions are indicated by fans. (**D**) Diagram illustrating conservation of the interface in yeast and human proteins. Interacting residues are shown as yellow (Ysh1) and orange (Mpe1) blocks. Hydrogen bonds or ionic interactions between side chains are depicted by solid grey lines, and 'mc' denotes that main chain atoms are involved. Dashed grey lines represent hydrophobic interactions. Equivalent interactions between conserved or similar residues in human orthologues CPSF73

(dark red) and RBBP6 (purple) are shown next to the relevant yeast residues. (**E**) Ribbon diagram showing structures of CPSF73 and RBBP6 docked together based on the Ysh1–Mpe1 structure. Details of the residues at the interaction interface are shown in two orthogonal views. Polar contacts are indicated by dashed yellow lines. These docking experiments suggest that this interface may be conserved in human CPSF73 and RBBP6, in agreement with a previously identified role for RBBP6 in pre-mRNA 3' end processing (Di Giammartino et al., 2014).

Figure S2 (opposite). Details of hydrogen-deuterium exchange (HDX), cross-link mass spectrometry and cryo EM. Related to Figure 2.

(**A**) Map of Ysh1, Mpe1 and Yjr141w sequence coverage in HDX experiments. Individual peptides are indicated by blue bars on the primary sequence. We were unable to purify isolated full-length Ysh1 so we instead performed a pair-wise analysis, comparing Ysh1 peptides from the Ysh1–Mpe1–Yjr141w trimer to equivalent peptides in the Ysh1–Mpe1 and Ysh1–Yjr141w dimers (**B**) Butterfly plot showing relative incorporation of deuterium into Ysh1 peptides at four different time-points, comparing Ysh1–Mpe1–Yjr141w (top) to Ysh1–Yjr141w (bottom). The corresponding difference plot indicates Ysh1 peptides that are affected by Mpe1 binding, and is shown in Figure 2B. (**C**) Butterfly plot as in (B) but comparing Ysh1–Mpe1–Yjr141w (top) to Ysh1–Mpe1 (bottom). The corresponding difference plot indicates Ysh1 peptides that are affected by Yjr141w binding, and is shown in Figure 2C. The relative fractional uptake of deuterium was generally higher in peptides from the Ysh1 C-terminal domain compared to peptides from the N-terminal catalytic domain, implying that the C-terminal domain is relatively disordered in the absence of other CPF subunits. (**D**) Plot from the Ysh1–Mpe1 sample showing relative incorporation of deuterium into Mpe1 peptides at four different time-points. Domains are color-coded as in Figure 1. This suggests that the only well-ordered regions are the N-terminal UBL and C-terminal RING domains. This flexibility may explain the crosslinks between the Ysh1 C-terminal domain and Mpe1 (Figure 2A), which are often observed in regions of proteins that are able to sample a larger conformational space. (**E**) Plot from the Ysh1–Yjr141w sample showing relative incorporation of deuterium into Yjr141w peptides at four different time-points. This did not reveal any obvious ordered domains. (B–E), triplicate data from four independent time-points are shown. Grey shading indicates the standard deviation of all charge states and replicates per peptide. (**F**) Residues indicated to be in close-proximity by cross-linking mass spectrometry experiments (Figure 2A) are mapped onto the crystal structure. Crosslinks are indicated by purple dashed lines. (**G**) Exploded view of the Ysh1–Mpe1 X-ray crystal structure. Residues highlighted in red are more protected from hydrogen-deuterium exchange when full-length Mpe1 is present (see Figure 2B). Residues 37–55 of Ysh1 interact directly with Mpe1 but it is not obvious from the crystal structure why residues 207–469 are protected by Mpe1 binding. We hypothesize that there are additional transient interactions with other parts of Mpe1 (that were absent in the construct used for crystallization) in this region of Ysh1. (**H**) Three orthogonal views showing the angular distribution of particles in the 3D cryo-EM reconstruction of the Ysh1–Mpe1–Yjr141w sample. A strongly preferred orientation is present. (**I**) Gold-standard Fourier shell correlation (FSC) curve for the Ysh1–Mpe1 cryo-EM map. (**J**) Point spread function (PSF) for the Ysh1–Mpe1 cryo-EM map, calculated in cryoEF (Naydenova and Russo, 2017). The efficiency of orientation distribution (E_{OD}) is 0.29, the mean PSF resolution is 4.7 Å, and the anisotropic PSF resolution is 2.1 Å – 8.51 Å (Naydenova and Russo, 2017).

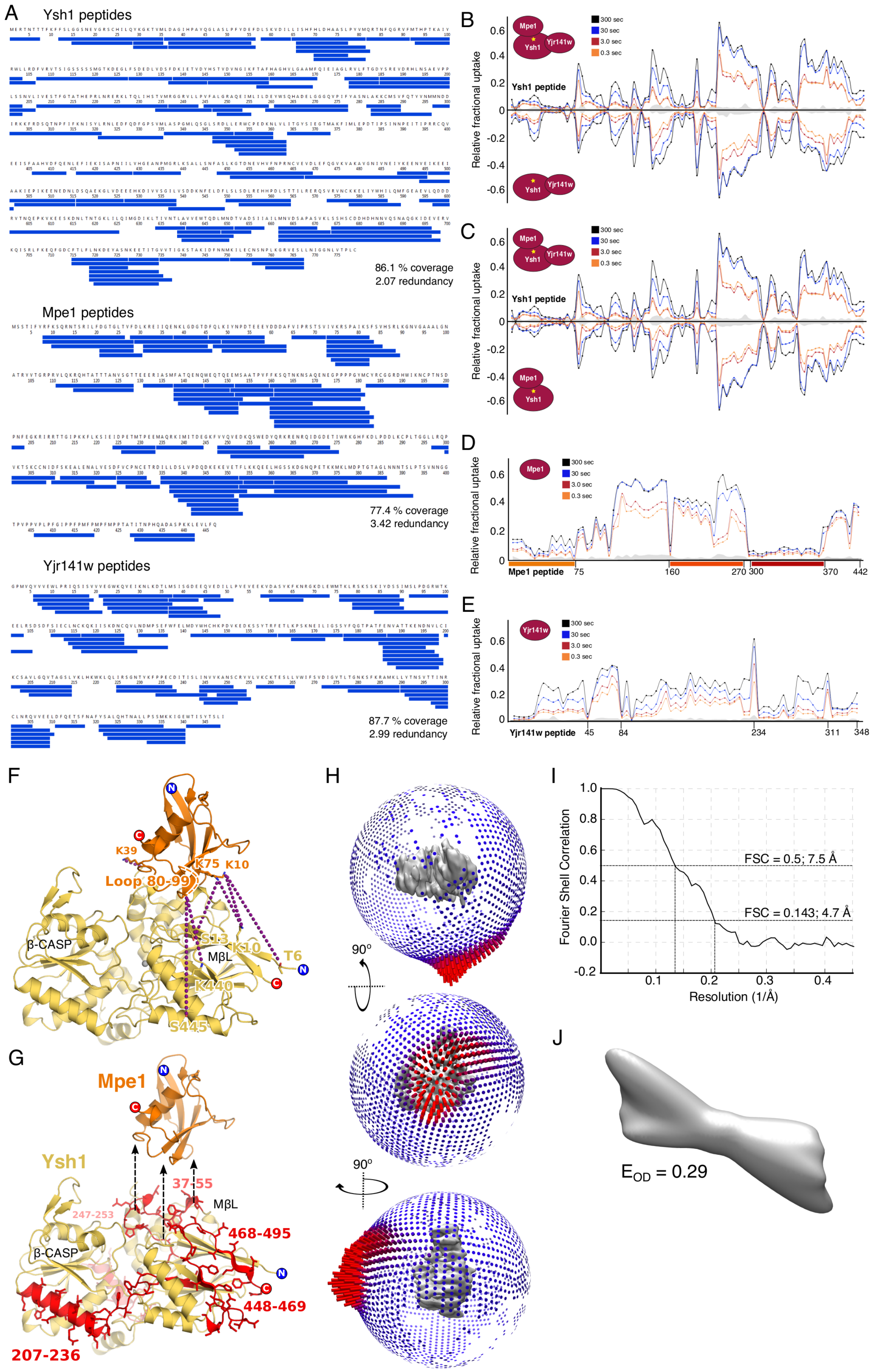
A
Ysh1 peptides
86.1 % coverage
2.07 redundancy
Mpe1 peptides
77.4 % coverage
3.42 redundancy
Yjr141w peptides
87.7 % coverage
2.99 redundancy
B
Mpe1
Ysh1
Yjr141w
300 sec
30 sec
3.0 sec
0.3 sec
Ysh1 peptide
Relative fractional uptake
C
D
Mpe1 peptide
E
Yjr141w peptide
F
K39
K75
K10
Loop 80-99
β-CASP
S13
MβL
T6
K440
S445
G
Mpe1
Ysh1
37-55
247-253
MβL
468-495
β-CASP
448-469
207-236
H
90°
I
Fourier Shell Correlation
FSC = 0.5; 7.5 Å
FSC = 0.143; 4.7 Å
Resolution (1/Å)
J
E_{OD} = 0.29

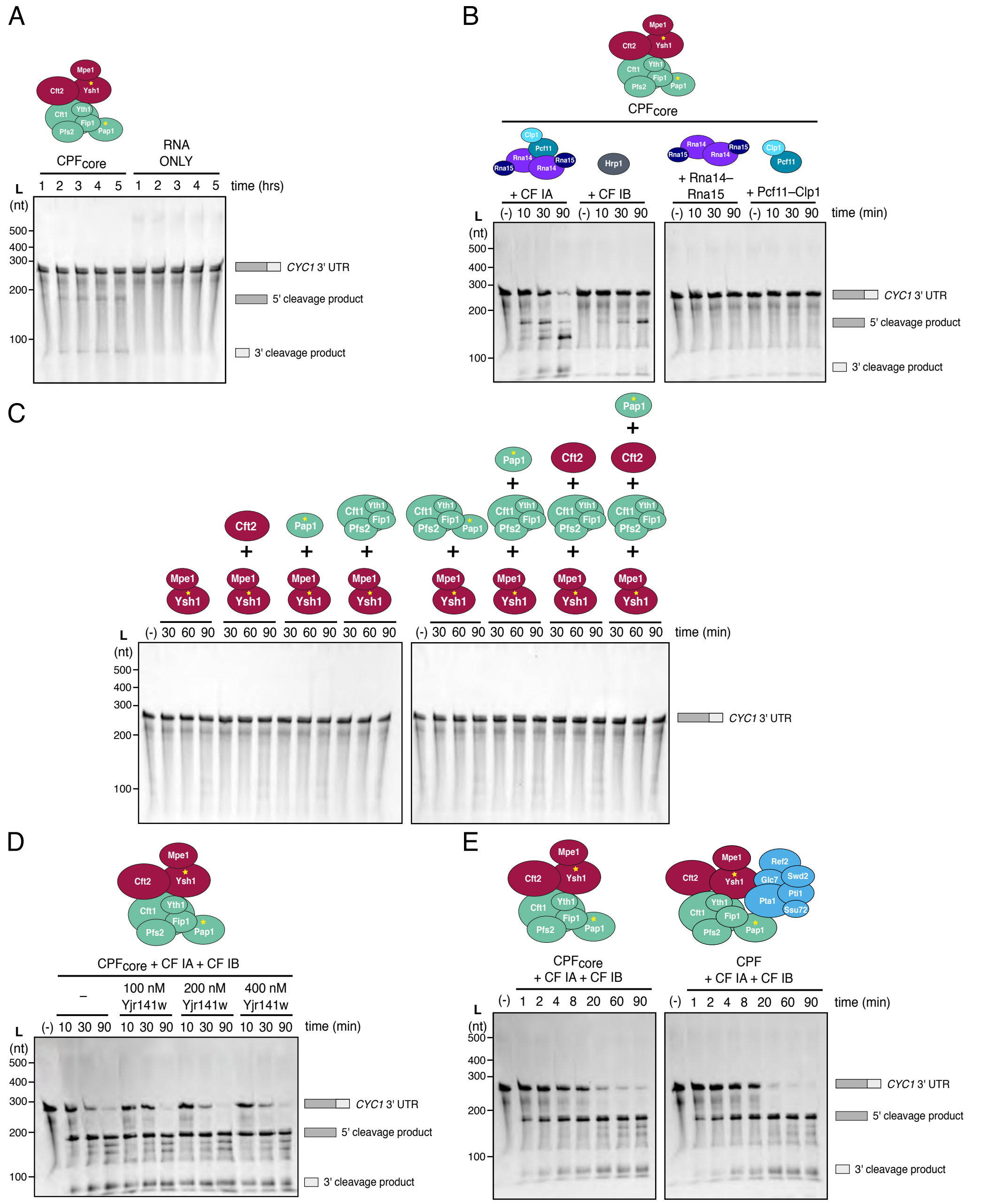

Figure S3. CPF_{core}, CF IA and CF IB are required for specific pre-mRNA cleavage *in vitro*. Related to Figure 3. All panels are Urea-PAGE analyses of cleavage assays performed using the *CYC1* 3'-UTR substrate. Negative control reactions (-) contained CF IA and/or CF IB but not proteins with enzymatic activity, and were incubated for the same duration as the longest time-point. (**A**) CPF_{core} is specific but has very low cleavage activity in the absence of CF IA and CF IB. We hypothesize that dynamics between the two domains of Ysh1 in solution may allow it to transiently sample the 'active' conformation with a wider substrate binding cleft. Atomic B-factors in the crystal structure imply that the β-CASP domain is more mobile, relative to the MβL domain. Incorporation of Ysh1 into CPF_{core} may shift the equilibrium towards a more open form via interaction with other subunits. In addition, the polymerase module provides RNA binding sites which are lacking in smaller Ysh1-containing complexes but are likely to further promote cleavage. (**B**) Comparison of the effects of CF IA, CF IB, Rna14–Rna15 and Pcf11–Clp1 on cleavage by CPF_{core}. CF IA has the strongest stimulatory effect, but additional cleavage of the upstream product occurs in the absence of CF IB. CF IB stimulates cleavage very weakly. The Rna14–Rna15 and Pcf11–Clp1 sub-complexes of CF IA do not stimulate cleavage. (**C**) Cleavage activity cannot be reconstituted by mixing equivalent molar amounts of independently expressed and purified sub-complexes, likely because they do not properly assemble *in vitro*. (**D**) Yjr141w does not substantially affect *in vitro* cleavage of *CYC1* by CPF_{core}. (**E**) CPF_{core} has very similar nuclease activity to the intact 14-subunit CPF purified from yeast (Casañal et al., 2017).

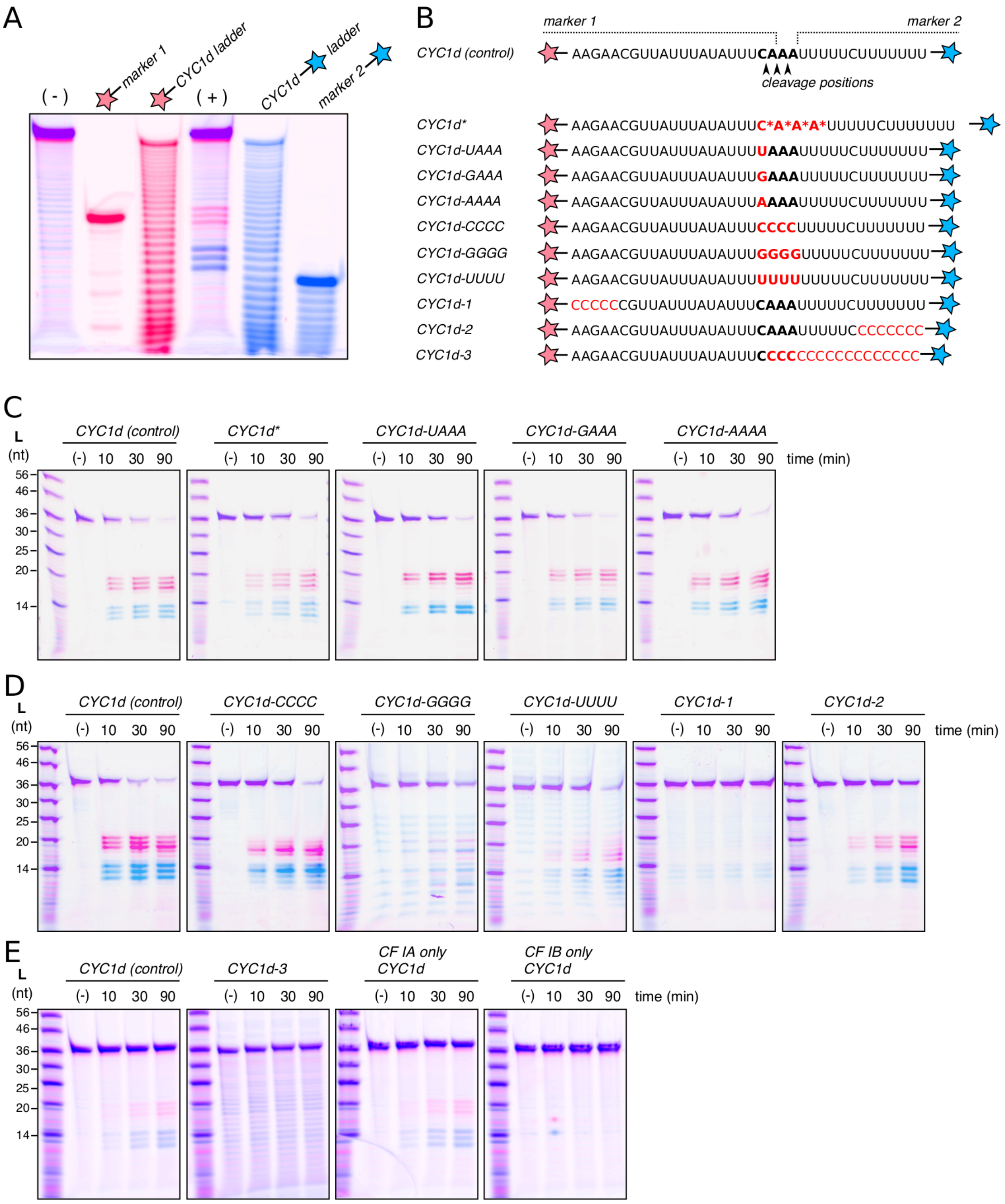

Figure S4. Effect of sequence changes on the cleavage of *CYC1* 3′-UTR short sequences. Related to Figure 4. (**A**) Urea-PAGE analysis of cleavage assays performed using the fluorescently-labelled *CYC1d* substrate, run alongside fluorescent fiducial RNA markers (see panel B) and alkaline hydrolysis ladders to determine the exact cleavage location. The lane marked (+) is loaded with a reaction containing CPF_{core}, CF IA and CF IB. The negative control reaction (-) contained CF IA and CF IB only. (**B**) Sequences of RNA substrates derived from the *CYC1* 3′-UTR. Each substrate carries both 5′-FAM and 3′-A647 labels (red and blue stars, respectively). The canonical cleavage site is highlighted in bold, and wedges denote the experimentally determined cleavage locations. Fiducial markers used in (A) are indicated above the sequence. Mutated nucleotides are in red. A star represents a phosphothioate bond, which is cleaved more slowly. (**C - E**) Urea-PAGE analysis of cleavage assays of the above substrates in the presence of CPF_{core}, CF IA and CF IB. Each gel should be compared to the positive control (left) from the same batch of experiments. The negative control reactions (-) contained CF IA and CF IB, but not CPF_{core} and were incubated for the duration of the longest time point.

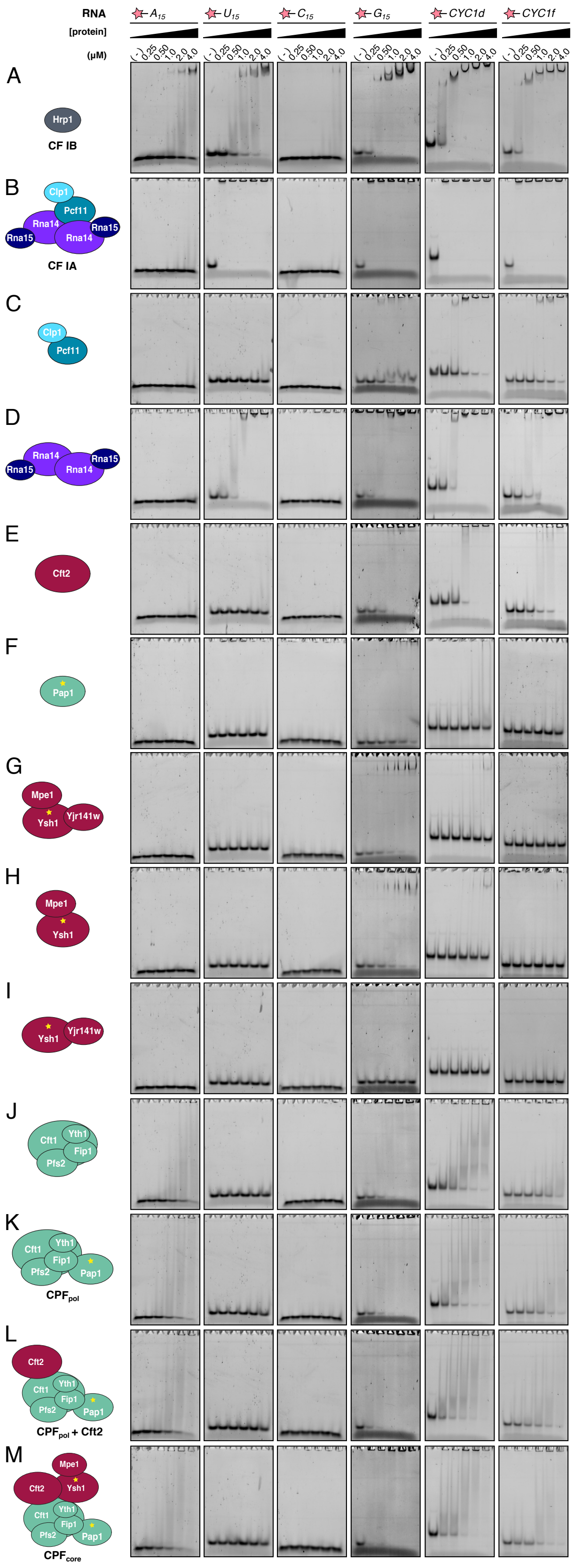

Figure S5. RNA binding by CPF_{core} and cleavage factor sub-components. Related to Figure 4 and Table 3. EMSAs performed with homopolymeric 15-mers of A, U, C and G, as well as *CYC1d* and *CYC1f* short RNAs. Proteins and sub-complexes tested were (**A**) CF IB, (**B**) CF IA, (**C**) Pcf11–Clp1, (**D**) Rna14–Rna15, (**E**) Cft2, (**F**) Pap1, (**G**) Ysh1–Mpe1–Yjr141w, (**H**) Ysh1–Mpe1, (**I**) Ysh1–Yjr141w, (**J**) CPF_{pol} lacking Pap1, (K) CPF_{pol}, (**L**) CPF_{pol}+Cft2 and (**M**) CPF_{core}. A summary of these results is in Table 3.

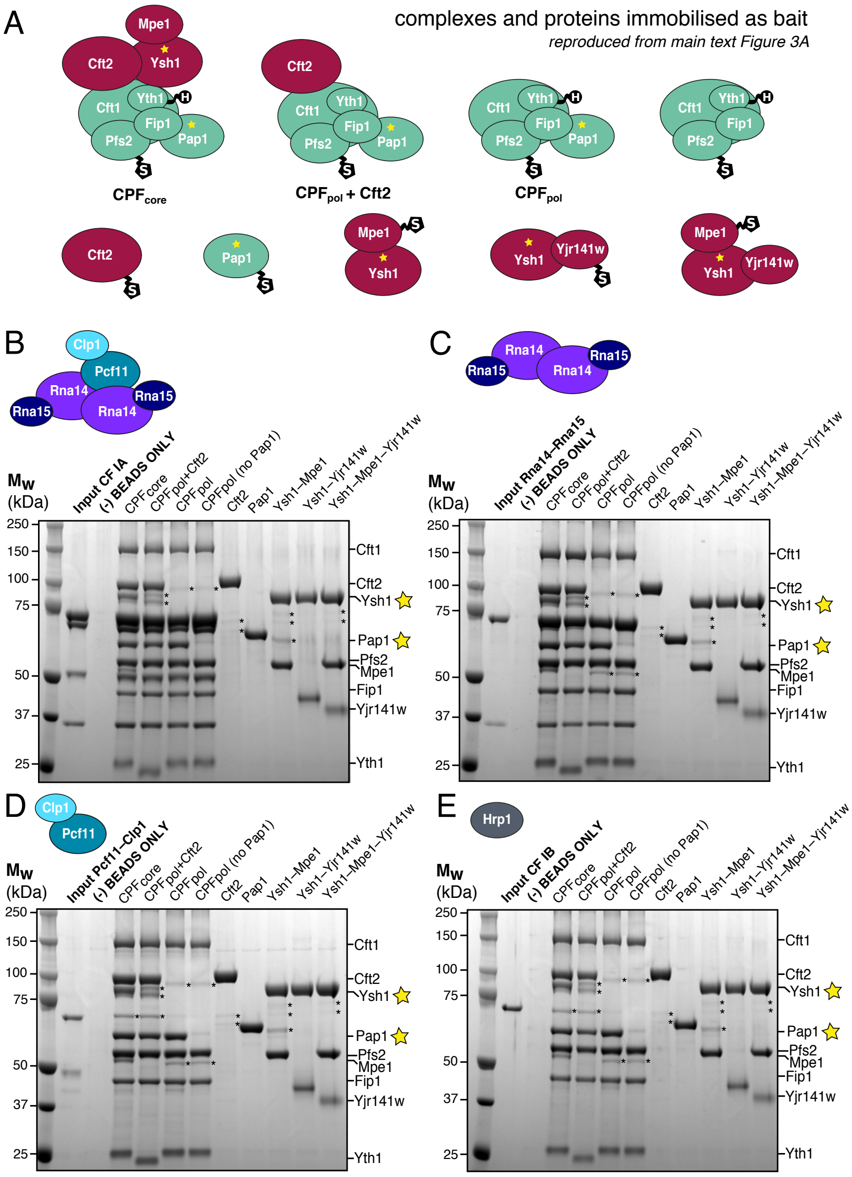

Figure S6. CF IA is recruited to CPF_{core} via interactions between Rna14–Rna15 and the polymerase module. Related to Figure 4.

Given that the accessory cleavage factors are indispensable for activation of the nuclease, we performed a series of pull-down experiments to map their ability to bind to different components of CPF_{core}. Pulldowns were performed using CPF proteins and complexes depicted in Figure 3A. To avoid misleading results caused by misfolded or aggregated proteins, we only used StrepII-tagged 'bait' proteins purified by anion exchange and size exclusion chromatography (Figure 3A). These were immobilized on strep-affinity resin and binding to untagged 'prey' complexes CF IA, Rna14–15, Pcf11–Clp1 and CF IB was analyzed. SDS-PAGE analyses of the bait-loaded resin after incubation with prey and four washes are shown. In all cases, the input lane contains the purified 'prey' proteins that were added. As a negative control (-) 'beads only' experiment, prey proteins were added to Streptactin resin without bait. Asterisks indicate contaminant proteins. CF IA and Rna14–15 bound tightly to complexes containing the polymerase module (with and without Pap1), as we had shown before (Casañal et al., 2017), but not to other proteins. In contrast, no CPF complexes interacted with Pcf11–Clp1 or CF IB. This indicates that the polymerase module is the primary component of core CPF that interacts directly with Rna14–Rna15 to recruit CF IA to the complex, and may also explain why CF IA activates cleavage to a greater extent than CF IB (Figure S3B).

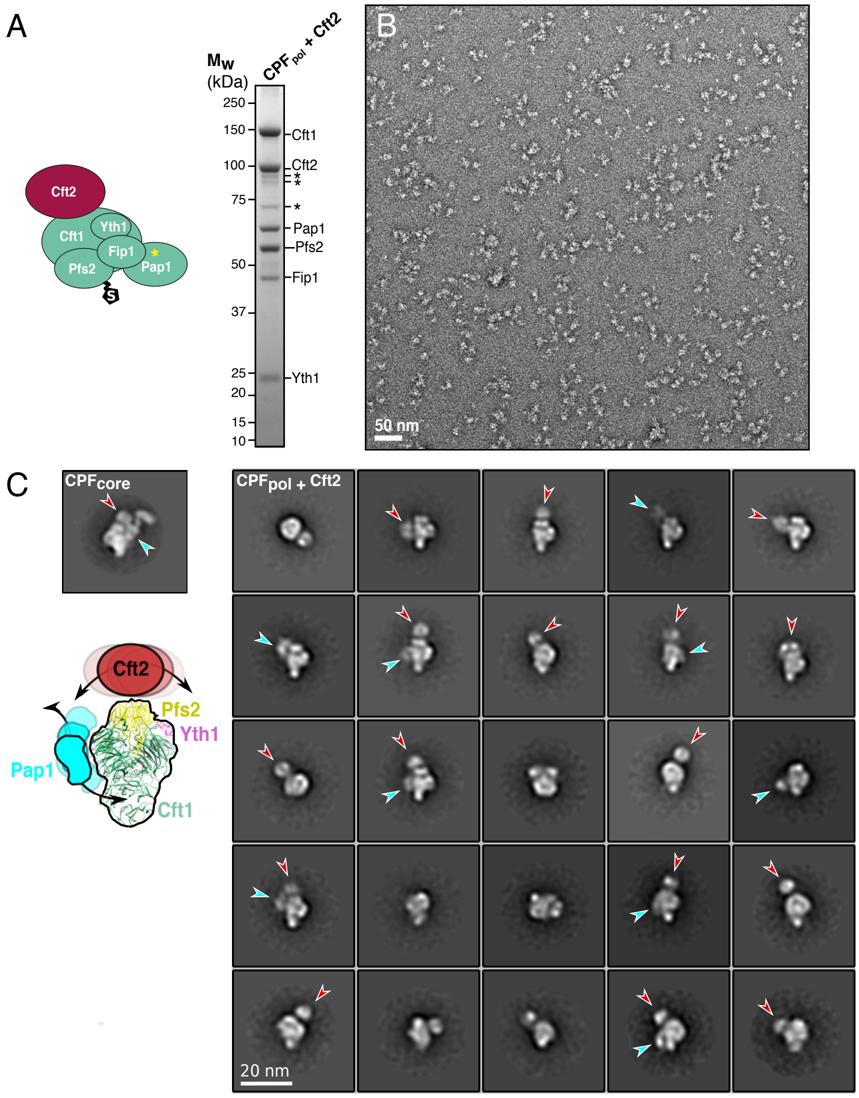

Figure S7. Mapping of Cft2 in CPF_{core} negative stain density. Related to Figure 5.
(**A**) To validate our low-resolution model of CPF_{core}, and in particular the positioning of Ysh1–Mpe1, we expressed and purified a version of CPF_{core} in which Ysh1 and Mpe1 were absent, i.e. the five-subunit polymerase module plus Cft2 (CPF_{pol}+Cft2). SDS-PAGE analysis of the purified complex is shown. Asterisks indicate contaminant proteins. (**B**) Representative negative stain micrograph of CPF_{pol}+Cft2 particles. (**C**) 2D class averages of CPF_{pol}+Cft2, compared to the predominant 21 nm particle present in CPF_{core} (see Figure 5C). This is consistent with our model for CPF_{core} because the hook-like globular density that we had assigned as Mpe1 and Ysh1 was missing. Omission of Ysh1 and Mpe1 appears to cause more conformational variability: the polymerase module is visible in each class with mobile extra densities. These are likely Cft2 (red arrowhead) and Pap1 (blue arrowhead), based on the shape of the densities. These data suggest that Mpe1 and Ysh1 have a role in stabilizing the overall assembly.

Table S1 Summary of Crosslink-MS experiments. Related to Figure 2.

				Mpe1:Ysh1		YJR141W:Ysh1		Mpe1:Ysh1:YJR141W	
**Protein 1**	**Res 1**	**Protein 2**	**Res2**	**BuUrBu**	**DSS**	**BuUrBu**	**DSS**	**BuUrBu**	**DSS**
Mpe1	10	Ysh1	6	X					
Mpe1	10	Ysh1	10	X				X	
Mpe1	39	Ysh1	119						X
Mpe1	75	YJR141W	60						X
Mpe1	75	Ysh1	4					X	
Mpe1	75	Ysh1	10	X	X			X	
Mpe1	75	Ysh1	13					X	
Mpe1	75	Ysh1	578		X				
Mpe1	77	Ysh1	440		X				
Mpe1	81	Ysh1	440					X	
Mpe1	82	Ysh1	440					X	
Mpe1	90	Ysh1	100		X				
Mpe1	90	Ysh1	228		X				
Mpe1	90	Ysh1	440	X	X			X	X
Mpe1	90	Ysh1	445	X				X	
Mpe1	90	Ysh1	508						X
Mpe1	90	Ysh1	701	X	X			X	
Mpe1	90	Ysh1	761		X				
Mpe1	114	Ysh1	116						X
Mpe1	114	Ysh1	117		X				
Mpe1	114	Ysh1	119		X				
Mpe1	114	Ysh1	226	X	X			X	
Mpe1	114	Ysh1	578		X				
Mpe1	114	Ysh1	583		X				
Mpe1	114	Ysh1	701	X	X				
Mpe1	114	Ysh1	704		X				
Mpe1	206	Ysh1	226	X	X			X	
Mpe1	216	YJR141W	141						X
Mpe1	263	Ysh1	226	X					
Mpe1	303	Ysh1	440					X	
Mpe1	303	Ysh1	451	X				X	
Mpe1	304	Ysh1	451		X				X
Mpe1	305	Ysh1	451		X				X
Mpe1	348	YJR141W	225						X
YJR141W	83	Ysh1	97				X		
YJR141W	84	Ysh1	96			X			
YJR141W	84	Ysh1	100			X			
YJR141W	97	Ysh1	761					X	
YJR141W	98	Ysh1	701					X	X
YJR141W	98	Ysh1	761						X
YJR141W	99	Ysh1	701				X		
YJR141W	99	Ysh1	761			X			
YJR141W	100	Ysh1	122				X		
YJR141W	100	Ysh1	123				X		
YJR141W	100	Ysh1	701			X	X		
YJR141W	153	Ysh1	701					X	X
YJR141W	155	Ysh1	701			X			
YJR141W	163	Ysh1	701					X	
YJR141W	165	Ysh1	701			X	X		

Table S2 Oligonucleotide sequences. Related to Figure 4 and STAR Methods.

RNAs	SOURCE / REF
CYC1 'long' substrate (used in activity assays):<br>5' –<br>GGGAGAACAGGCCCCUUUUCCUUUGUCGAUAUCAUGUAAUUAGUUAUGUCACGCUUA<br>CAUUCACGCCCUCCUCCCACAUCCGCUCUAACCGAAAAGGAAGGAGUUAGACAACCUGA<br>AGUCUAGGUCCCUAUUUAUUUUUUUUAUAGUUAUGUUAGUAUUAAGAACGUUAUUU<br>AUAUUUCAAAUUUUUCUUUUUUUUCUGUACAAACGCGUGUACGCAUGUAACAUUAUA<br>CUGAAAACCUUGCUUGAGAAGGUUUUGGGA – 3'	Casañal et al., 2017
CYC1a 'short' substrate (used in activity assays):<br>5' – FAM<br>UUUAUAGUUAUGUUAGUAUUAAGAACGUUAUUUAUAUUUCAAAUUUUUCUUUUUUU<br>– A647 3'	IDT fluorescent oligo
CYC1b 'short' substrate (used in activity assays):<br>5' – FAM<br>UGUUAGUAUUAAGAACGUUAUUUAUAUUUCAAAUUUUUCUUUUUUUUCUGUACAGA<br>– A647 3'	IDT fluorescent oligo
CYC1c 'short' substrate (used in activity assays):<br>5' – FAM AAGAACGUUAUUUAUAUUUCAAAUUUUUCUUUUUUUUCUGUACAGA –<br>A647 3'	IDT fluorescent oligo
CYC1d 'short' substrate (used in activity assays):<br>5' – FAM AAGAACGUUAUUUAUAUUUCAAAUUUUUCUUUUUUU – A647 3'	IDT fluorescent oligo
CYC1d* 'short' substrate (used in activity assays, * indicates phosphothioate bonds)<br>5' – FAM AAGAACGUUAUUUAUAUUUC*A*A*A*UUUUUCUUUUUUU – A647 3'	IDT fluorescent oligo
CYC1d-UAAA 'short' substrate (used in activity assays):<br>5' – FAM AAGAACGUUAUUUAUAUUUUAAAUUUUUCUUUUUUU – A647 3'	IDT fluorescent oligo
CYC1d-GAAA 'short' substrate (used in activity assays):<br>5' – FAM AAGAACGUUAUUUAUAUUUGAAAUUUUUCUUUUUUU – A647 3'	IDT fluorescent oligo
CYC1d-AAAA 'short' substrate (used in activity assays):<br>5' – FAM AAGAACGUUAUUUAUAUUUAAAAUUUUUCUUUUUUU – A647 3'	IDT fluorescent oligo
CYC1d-CCCC 'short' substrate (used in activity assays):<br>5' – FAM AAGAACGUUAUUUAUAUUUCCCCUUUUUCUUUUUUU – A647 3'	IDT fluorescent oligo
CYC1d-GGGG 'short' substrate (used in activity assays):<br>5' – FAM AAGAACGUUAUUUAUAUUUGGGGUUUUUCUUUUUUU – A647 3'	IDT fluorescent oligo
CYC1d-UUUU 'short' substrate (used in activity assays):<br>5' – FAM AAGAACGUUAUUUAUAUUUUUUUUUUUUCUUUUUUU – A647 3'	IDT fluorescent oligo
CYC1d-1 'short' substrate (used in activity assays):<br>5' – FAM CCCCCCGUUAUUUAUAUUUCAAAUUUUUCUUUUUUU – A647 3'	IDT fluorescent oligo
CYC1d-2 'short' substrate (used in activity assays):<br>5' – FAM AAGAACGUUAUUUAUAUUUCAAAUUUUUCCCCCCCC – A647 3'	IDT fluorescent oligo

CYC1d-3 ‘short’ substrate (used in activity assays):<br>5’ – FAM AAGAACGUUAUUUAUAUUUCCCCCCCCCCCCCCCC – A647 3’	IDT fluorescent oligo
CYC1e ‘short’ substrate (used in activity assays):<br>5’ – FAM AAGAACGUUAUUUAUAUUUCAAAUUUUUCU A647 – 3’	IDT fluorescent oligo
CYC1f ‘short’ substrate (used in activity assays):<br>5’ – FAM CGUUAUUUAUAUUUCAAAUUUUUCU A647 – 3’	IDT fluorescent oligo
CYC1g ‘short’ substrate (used in activity assays):<br>5’ – FAM UUUAUAUUUCAAAUUUUUCU A647 – 3’	IDT fluorescent oligo
CYC1h ‘short’ substrate (used in activity assays):<br>5’ – FAM UUUUUCAAAUUUUU A647 – 3’	IDT fluorescent oligo
**A_{15} probe** (used in EMSA):<br>5’ – FAM AAAAAAAAAAAAAAA – 3’	IDT fluorescent oligo
**U_{15} probe** (used in EMSA):<br>5’ – FAM UUUUUUUUUUUUUUU – 3’	IDT fluorescent oligo
**C_{15} probe** (used in EMSA):<br>5’ – FAM CCCCCCCCCCCCCCC – 3’	IDT fluorescent oligo
**G_{15} probe** (used in EMSA):<br>5’ – FAM GGGGGGGGGGGGGGG – 3’	IDT fluorescent oligo
CYC1d **probe** (used in EMSA):<br>5’ – FAM AAGAACGUUAUUUAUAUUUCAAAUUUUUCUUUUUU – 3’	IDT fluorescent oligo
CYC1f **probe** (used in EMSA):<br>5’ – FAM CGUUAUUUAUAUUUCAAAUUUUUCU – 3’	IDT fluorescent oligo

**DNA Oligonucleotides**	**SOURCE / REF**
**Pap1_F (**PCR primer for cloning)<br>5’ – AATTGGATCCATGAGCAGCCAGAAAGTTTTT – 3’	Sigma-Aldrich<br>VC00021
**Pap1_R** (PCR primer for cloning)<br>5’ – AATTCTCGAGATTAACATCAACTGCTGCGG – 3’	Sigma-Aldrich<br>VC00021
**Cft2_F** (PCR primer for cloning)<br>5’ – AATTGGATCCATGACCTATAAATACAATTG – 3’	Sigma-Aldrich<br>VC00021
**Cft2_R** (PCR primer for cloning)<br>5’ – AATTCTCGAGAATTTTGGCCAGCATATCTG – 3’	Sigma-Aldrich<br>VC00021
**biGBac_pIDS_CasI_F** (PCR primer for cloning)<br>5’ – AACGCTCTATGGTCTAAAGATTTAAATGGCACCTAGGTATCGATACTAGTATAC – 3’	Sigma-Aldrich<br>VC00021
**biGBac_pIDS_CasI_R** (PCR primer for cloning)<br>5’ – AACCCCGATTGAGATATAGATTTATTTAAATGTACCCGTAGTGGCTATG – 3’	Sigma-Aldrich<br>VC00021
**Ysh1-N_F** (PCR primer for cloning)<br>5’ – AATTGGATCCCTCGAGATGGAACGTACCAATACCAC – 3’	Sigma-Aldrich<br>VC00021

**Ysh1-N_R** (PCR primer for cloning)<br>5' – AATTTCTAGAGGTACCTTAACCCTGAAATTCCAGATCCA – 3'	Sigma-Aldrich<br>VC00021
**Ysh1-C_F** (PCR primer for cloning)<br>5' – AATTGGATCCCTCGAGATGGTTAAAGTTGCAAAAGCCGT – 3'	Sigma-Aldrich<br>VC00021
**Ysh1-C_R** (PCR primer for cloning)<br>5' – AATTTCTAGAGGTACCTTAACACAGCGGTGTAACCA – 3'	Sigma-Aldrich<br>VC00021
**Mpe1-1_F** (PCR primer for cloning)<br>5' – AATTGGATCCATGAGCAGCACCATCTTTTATC – 3'	Sigma-Aldrich<br>VC00021
**Mpe1-1_R** (PCR primer for cloning)<br>5' – AATTCTCGAGCTGATTACCATCTTTGCTGC – 3'	Sigma-Aldrich<br>VC00021
**Mpe1-2_F** (PCR primer for cloning)<br>5' – AATTGGATCCATGAGCAGCACCATCTTTTATC – 3'	Sigma-Aldrich<br>VC00021
**Mpe1-2_R** (PCR primer for cloning)<br>5' – AATTCTCGAGATCAATCTGACGATTTTCAC – 3'	Sigma-Aldrich<br>VC00021
**Mpe1-3_F** (PCR primer for cloning)<br>5' – AATTGGATCCATGAGCAGCACCATCTTTTATC – 3'	Sigma-Aldrich<br>VC00021
**Mpe1-3_R** (PCR primer for cloning)<br>5' – AATTCTCGAGGAAAAAAACCGGAGTTGCTGC – 3'	Sigma-Aldrich<br>VC00021
**Mpe1-4_F** (PCR primer for cloning)<br>5' – AATTGGATCCATGAAAAGCTTTAGCGTTCATAGC – 3'	Sigma-Aldrich<br>VC00021
**Mpe1-4_R** (PCR primer for cloning)<br>5' – AATTCTCGAGTTTTTTCGGGCTTGCATCTG – 3'	Sigma-Aldrich<br>VC00021
**Mpe1-5_F** (PCR primer for cloning)<br>5' – AATTGGATCCATGAAAAGCCAGACCAATAAAAAC – 3'	Sigma-Aldrich<br>VC00021
**Mpe1-5_R** (PCR primer for cloning)<br>5' – AATTCTCGAGTTTTTTCGGGCTTGCATCTG – 3'	Sigma-Aldrich<br>VC00021
